# Poisson traces, D-modules, and symplectic resolutions

**DOI:** 10.1007/s11005-017-1024-1

**Published:** 2017-12-05

**Authors:** Pavel Etingof, Travis Schedler

**Affiliations:** 1 Massachusetts Institute of Technology, Cambridge, MA USA; 2 Imperial College London, London, UK

**Keywords:** Hamiltonian flow, Complete intersections, Milnor number, D-modules, Poisson homology, Poisson varieties, Poisson homology, Poisson traces, Milnor fibration, Calabi–Yau varieties, Deformation quantization, Kostka polynomials, Symplectic resolutions, Twistor deformations, 14F10, 37J05, 32S20, 53D55

## Abstract

We survey the theory of Poisson traces (or zeroth Poisson homology) developed by the authors in a series of recent papers. The goal is to understand this subtle invariant of (singular) Poisson varieties, conditions for it to be finite-dimensional, its relationship to the geometry and topology of symplectic resolutions, and its applications to quantizations. The main technique is the study of a canonical D-module on the variety. In the case the variety has finitely many symplectic leaves (such as for symplectic singularities and Hamiltonian reductions of symplectic vector spaces by reductive groups), the D-module is holonomic, and hence, the space of Poisson traces is finite-dimensional. As an application, there are finitely many irreducible finite-dimensional representations of every quantization of the variety. Conjecturally, the D-module is the pushforward of the canonical D-module under every symplectic resolution of singularities, which implies that the space of Poisson traces is dual to the top cohomology of the resolution. We explain many examples where the conjecture is proved, such as symmetric powers of du Val singularities and symplectic surfaces and Slodowy slices in the nilpotent cone of a semisimple Lie algebra. We compute the D-module in the case of surfaces with isolated singularities and show it is not always semisimple. We also explain generalizations to arbitrary Lie algebras of vector fields, connections to the Bernstein–Sato polynomial, relations to two-variable special polynomials such as Kostka polynomials and Tutte polynomials, and a conjectural relationship with deformations of symplectic resolutions. In the appendix we give a brief recollection of the theory of D-modules on singular varieties that we require.

## Introduction

**1.1.** This paper gives an introduction to the theory of traces on Poisson algebras developed by the authors in a series of recent papers [13, 14, 20, 22–25, 50]. It is based on two minicourses given by the authors at Cargese (2014) and ETH Zurich (2016).

Let *A* be a Poisson algebra over $\mathbf C$, for example, $A={\mathcal {O}}(X)$, where *X* is an affine Poisson variety. A *Poisson trace* on *A* is a linear functional $A \rightarrow \mathbf C$ which annihilates $\{A, A\}$, i.e., a Lie algebra character of *A*. The space of such traces is the dual, ${\textsf {HP}}_0(A)^*$, to the *zeroth Poisson homology*, ${\textsf {HP}}_0(A) := A /\{A, A\}$, the abelianization of *A* as a Lie algebra (where $\{A,A\}$ denotes the $\mathbf C$-linear span of Poisson brackets of functions).

The space ${\textsf {HP}}_0(A)$ is an important but subtle invariant of *A*. For example, it is a nontrivial question when ${\textsf {HP}}_0(A)$ is finite dimensional. Indeed, even in the simple case $A=\mathcal {O}(V)^G$, where *V* is a symplectic vector space and *G* a finite group of symplectic transformations of *V*, finite dimensionality of ${\textsf {HP}}_0(A)$ used to be a conjecture due to Alev and Farkas [1]. It is even harder to find or estimate the dimension of ${\textsf {HP}}_0(A)$; this is, in general, unknown even for $A=\mathcal {O}(V)^G$.

The first main result of the paper is a wide generalization of the Alev–Farkas conjecture, stating that ${\textsf {HP}}_0(A)$ is finite dimensional if $X:={\textsf {Spec}}\,A$ is a Poisson variety (or, more generally, scheme of finite type) with finitely many symplectic leaves. Namely, the Alev–Farkas conjecture is obtained in the special case $X=V/G$. A more general example is $X=Y/G$ where *Y* is an affine symplectic variety, and *G* is a finite group of automorphisms of *Y* (such as symmetric powers of affine symplectic varieties). But there are many other examples, such as Hamiltonian reductions of symplectic vector spaces by reductive groups acting linearly and affine symplectic singularities (which includes nilpotent cones and Slodowy slices, hypertoric varieties). This result can be applied to show that any quantization of such a variety has finitely many irreducible finite-dimensional representations (at most $\dim {\textsf {HP}}_0(\mathcal {O}(X))$).

The proof of this result is based on the theory of D-modules (as founded in 1970–1971 by Bernstein and Kashiwara in, e.g., [7, 41]). Namely, we define a canonical D-module on *X*, denoted *M*(*X*), such that $HP_0(\mathcal {O}(X))$ is the underived direct image $H^0 \pi _{*}M(X)$ under the map $\pi : X\rightarrow \mathrm {pt}$. Namely, if $i: X\hookrightarrow V$ is a closed embedding of *X* into an affine space, then *M*(*X*), regarded as a right $\mathcal {D}(V)$-module supported on *i*(*X*), is the quotient of $\mathcal {D}(V)$ by the right ideal generated by the equations of *i*(*X*) in *V* and by Hamiltonian vector fields on *X*. We show that if *X* has finitely many symplectic leaves, then *M*(*X*) is a holonomic D-module (which extends well-known results on group actions on varieties). Then, a standard result in the theory of D-modules implies that ${\textsf {HP}}_0({\mathcal {O}}(X))=H^0\pi _{*}M(X)$ is finite dimensional.

In fact, this method can be applied to a more general problem, when we have an affine variety *X* acted upon by a Lie algebra $\mathfrak {g}$. We say that *X* has finitely many $\mathfrak {g}$-leaves if *X* admits a finite stratification with locally closed connected strata $X_i$ (called $\mathfrak {g}$-leaves) which carry a transitive action of $\mathfrak {g}$ (i.e., $\mathfrak {g}$ surjects to each tangent space of $X_i$). Then, we show that if *X* has finitely many $\mathfrak {g}$-leaves, then the space of coinvariants ${{\mathcal {O}}}(X)/\lbrace { \mathfrak {g},{\mathcal {O}}(X)\rbrace }$ is finite dimensional. The previous result on Poisson varieties is then recovered if $\mathfrak {g}$ is ${{\mathcal {O}}}(X)$. The proof of this more general result is similar: we define a canonical D-module $M(X,\mathfrak {g})$, obtained by dividing $\mathcal {D}(V)$ by the equations of *X* and by $\mathfrak {g}$, and show that its underived direct image $H^0 \pi _{*}M(X,\mathfrak {g})$ to a point is ${{\mathcal {O}}}(X)/\lbrace { \mathfrak {g},{\mathcal {O}}(X)\rbrace }$ and that it is holonomic if *X* has finitely many $\mathfrak {g}$-leaves. In this setting, the result is well known if the action of $\mathfrak {g}$ integrates to an action of a connected algebraic group *G* with ${\textsf {Lie}}G = \mathfrak {g}$, and in this case $M(X,\mathfrak {g})$ is in fact regular (see, e.g., [57, Lemma 1], [40, Theorem 4.1.1], [35, Section 5]); cf. Remark 6.10 below.

Moreover, the definition of $M(X,\mathfrak {g})$ makes sense when *X* is not necessarily affine, and $\mathfrak {g}$ is a presheaf of Lie algebras on *X* which satisfies a ${\mathcal {D}}$-localizability condition: $\mathfrak {g}(U){\mathcal {D}}(U')=\mathfrak {g}(U'){\mathcal {D}}(U')$ for any open affines $U'\subset U\subset X$. This condition is satisfied, in particular, when *X* is Poisson and $\mathfrak {g}={{\mathcal {O}}}(X)$. Furthermore, it is interesting to consider the full direct image $\pi _*M(X,\mathfrak {g})$. Its cohomology $H^i(\pi _*M(X,\mathfrak {g}))$ then ranges between $i=-\dim X$ and $i=\dim X$, and we call it the $\mathfrak {g}$-de Rham homology of *X*. If $\mathfrak {g}={\mathcal {O}}(X)$ for a Poisson variety *X*, we call this cohomology the Poisson-de Rham homology of *X*, denoted ${\textsf {HP}}_{-i}^{DR}(X)$. For instance, if *X* is affine, then ${\textsf {HP}}_0^{DR}(X) \cong {\textsf {HP}}_0({\mathcal {O}}(X))$.

The rest of the paper is dedicated to the study of the D-module *M*(*X*) and the Poisson-de Rham homology (in particular, ${\textsf {HP}}_0({\mathcal {O}}(X))$ when *X* is affine) for specific examples of Poisson varieties *X*. One of the main cases of interest is the case when *X* admits a symplectic resolution $\rho : {\widetilde{X}}\rightarrow X$. In this case we conjecture that $M(X)\cong \rho _*\Omega _{{\widetilde{X}}}$. Namely, since $\rho$ is known to be semismall, $\rho _*\Omega _X$ is a semisimple regular holonomic D-module (concentrated in the cohomological degree 0), and one can show that it is isomorphic to the semisimplification of a quotient of *M*(*X*), so the conjecture is that this quotient is by zero and that *M*(*X*) is semisimple. This conjecture implies that $\dim {\textsf {HP}}_0({\mathcal {O}}(X))=\dim H^{\dim X}({\widetilde{X}},\mathbf C )$ for affine *X*, and, more generally, ${\textsf {HP}}_i^{DR}(X)\cong H^{\dim X-i}({\widetilde{X}},\mathbf C )$.

We discuss a number of cases when this conjecture is known: symmetric powers of symplectic surfaces with Kleinian singularities, Slodowy slices of the nilpotent cone of a semisimple Lie algebra, and hypertoric varieties. However, the conjecture is open for an important class of varieties admitting a symplectic resolution: Nakajima quiver varieties.

It turns out that an explicit calculation of *M*(*X*) and the Poisson-de Rham homology of *X* (in particular, ${\textsf {HP}}_0(\mathcal {O}(X))$ for affine *X*) is sometimes possible even when *X* does not admit a symplectic resolution. Namely, one can compute these invariants for symmetric powers of symplectic varieties of any dimension, and for arbitrary complete intersection surfaces in $\mathbf C ^n$ with isolated singularities. We discuss these calculations at the end of the paper. For example, it is interesting to compute *M*(*X*) when *X* is the cone of a smooth curve *C* of degree *d* in $\mathbf P ^2$. Recall that the genus of *C* is $(d-1)(d-2)/2$, and the Milnor number of *X* is $\mu =(d-1)^3$. We show that $M(X) \cong \delta ^{\mu -g}\oplus M(X)_{\text {ind}}$, where $M(X)_{\text {ind}}$ is an indecomposable D-module containing $\delta ^{2g}$, such that $M(X)_{\text {ind}}/\delta ^{2g}$ is an indecomposable extension of $\delta ^g$ by the intersection cohomology D-module ${\text {IC}}(X)$. (Here $\delta$ is the $\delta$-function D-module supported at the vertex of *X*.)

The structure of the paper is as follows. In Sect. 2 we define $\mathfrak {g}$-leaves of a variety with an $\mathfrak {g}$-action, state the finite dimensionality theorem for coinvariants on varieties with finitely many leaves, and give a number of examples (notably in the Poisson case). In Sect. 3 we apply this theorem to proving that quantizations of Poisson varieties with finitely many leaves have finitely many irreducible finite-dimensional representations. In Sect. 4 we prove the finite dimensionality theorem using D-modules. In Sect. 5 we define the $\mathfrak {g}$-de Rham and Poisson-de Rham homology. In Sect. 6 we discuss the conjecture on the Poisson-de Rham homology of Poisson varieties admitting a symplectic resolution. In Sect. 7 we discuss Poisson-de Rham homology of symmetric powers. In Sect. 8 we discuss the structure of *M*(*X*) when *X* is a complete intersection with isolated singularities. In Sect. 9 we discuss weights on the Poisson-de Rham homology (and hence ${\textsf {HP}}_0$) of cones and state an enhancement of the aforementioned conjecture in this case which incorporates weights. Finally, in the appendix we review background on D-modules used in the body of the paper.

### Notation

Fix throughout an algebraically closed field $\mathbf k$ of characteristic zero (the algebraically closed hypothesis is for convenience and is inessential); we will restrict to $\mathbf k =\mathbf C$ in Sects. 6–9. We work with algebraic varieties over $\mathbf k$, which we take to mean reduced separated schemes of finite type over $\mathbf k$; we frequently work with affine varieties. (However, see Remarks 4.16 and 4.17 for some analogous results in the $C^\infty$ and complex analytic settings.) We will let $\mathcal {O}_X$ denote the structure sheaf of *X*, and for $U \subseteq X$, we let $\mathcal {O}(U) := \Gamma (U,\mathcal {O}_X)$. Recall that there is a coherent sheaf $T_X$, called the tangent sheaf, on *X* with the property that, for every affine open subset $U \subseteq X$, $\Gamma (U,T_X) \cong {\text {Der}}(\mathcal {O}(U))$, the Lie algebra of $\mathbf k$-algebra derivations of $\mathcal {O}(U)$, which by definition are the vector fields on *U*. (In the literature, $T_X$ is often defined as ${\text {Hom}}_{\mathcal {O}_X}(\Omega ^1_X, T_X)$ where $\Omega ^1_X$ is the sheaf of Kähler differentials. In some references $T_X$ is restricted to the case that *X* is smooth, which implies that $T_X$ is a vector bundle, but in general $T_X$ need not be locally free; see, e.g., [54, pp. 88–89] for a reference for $T_X$ in general.) Let ${\text {Vect}}(X):=\Gamma (X,T_X)$, which is a Lie algebra whose elements are called global vector fields on *X*, and which is a module over the ring $\mathcal {O}(X)$ of global functions.

## Finite dimensionality of coinvariants under flows and zeroth Poisson homology

**2.1.** Let $\mathfrak {g}$ be a Lie algebra over $\mathbf k$ and *X* be an affine variety over $\mathbf k$ (which very often will be singular). Suppose that $\mathfrak {g}$ acts on *X*, i.e., we have a Lie algebra homomorphism $\alpha : \mathfrak {g} \rightarrow {\text {Vect}}(X)$. (We can take $\mathfrak {g} \subseteq {\text {Vect}}(X)$ and $\alpha$ to be the inclusion if desired.) In this case, $\mathfrak {g}$ acts on $\mathcal {O}(X)$ by derivations, and we can consider the coinvariant space, $\mathcal {O}(X)_\mathfrak {g}:= \mathcal {O}(X)/\mathfrak {g}\cdot \mathcal {O}(X)$, which is also denoted by $H_0(\mathfrak {g}, \mathcal {O}(X))$. (It is the zeroth Lie homology of $\mathfrak {g}$ with coefficients in $\mathcal {O}(X)$.) We begin with a criterion for this to be finite dimensional, which is the original motivation for the results of this note.

Say that the action is transitive if the map $\alpha _x: \mathfrak {g}\rightarrow T_x X$ is surjective for all $x \in X$. It is easy to see that if the action is transitive, then *X* is smooth, since ${\text {rk}}\alpha _x$ is upper semicontinuous, while $\dim T_x X$ is lower semicontinuous (in other words, generically $\alpha$ has maximal rank and *X* is smooth, so if $\alpha _x$ is surjective for all *x*, then $\dim T_x X = {\text {rk}}\alpha _x$ is constant for all *x*, and hence *X* is smooth).

### Example 2.1

If $\mathfrak {g}= {\text {Vect}}(X)$, then $\mathfrak {g}$ acts transitively if and only if *X* is smooth. (Since it is clear that, if *X* is smooth affine, then global vector fields restrict to $T_xX$ at every $x \in X$.) Moreover, if *X* is singular, it is a theorem of A. Seidenberg [53] that $\mathfrak {g}$ is tangent to the set-theoretic singular locus (although this would be false in characteristic *p*).

In the case that $\mathfrak {g}$ does not act transitively, one can attempt to partition *X* into leaves where it does act transitively. This motivates

### Definition 2.2

A $\mathfrak {g}$-leaf on *X* is a maximal locally closed connected subvariety $Z \subseteq X$ such that, for all $z \in Z$, the image of $\alpha _z$ is $T_z Z$.

Note that a $\mathfrak {g}$-leaf is smooth since the tangent spaces $T_z Z$ have constant dimension, and hence, it is irreducible. Thus, a $\mathfrak {g}$-leaf is a maximal locally closed irreducible subvariety *Z* such that $\mathfrak {g}$ preserves *Z* and acts transitively on it.

### Remark 2.3

Note that two distinct $\mathfrak {g}$-leaves are disjoint, since if $Z_1, Z_2$ are two intersecting leaves, then the union $Z=Z_1 \cup Z_2$ is another connected locally closed set such that the image of $\alpha _z$ is $T_z Z$ for all $z \in Z$; by maximality $Z_1=Z=Z_2$. Therefore, if *X* is a union of $\mathfrak {g}$-leaves, then this union is disjoint and the decomposition is canonical.

On the other hand, it is not always true that *X* is a union of its leaves. Indeed, *X* can sometimes have no leaves at all. For example, let $X=(\mathbf C ^\times )^2=\,{\textsf {Spec}}\mathbf C [x,x^{-1},y,y^{-1}]$ and $\mathfrak {g}= \mathbf C \cdot \xi$ for $\xi$ a global vector field which is not algebraically integrable, such as $\xi =x\partial _x - c y \partial _y$ for *c* irrational. If we work instead in the analytic setting, then locally there do exist analytic $\mathfrak {g}$-leaves, which in the example are the local level sets of $x^c y$; but these are not algebraic (and do not even extend to global analytic leaves), as we are requiring.

### Theorem 2.4

([20, Theorem 3.1], [25, Theorem 1.1]) If *X* is a union of finitely many $\mathfrak {g}$-leaves, then the coinvariant space $\mathcal {O}(X)_\mathfrak {g}= \mathcal {O}(X) / \mathfrak {g}\cdot \mathcal {O}(X)$ is finite-dimensional.

### Remark 2.5

Let $X_i:=\{x \in X \mid {\text {rk}}\alpha _x = i\}$ be the locus where the infinitesimal action of $\mathfrak {g}$ restricts to an *i*-dimensional subspace of the tangent space. This is a locally closed subvariety. If it has dimension *i*, then its connected components are the leaves of dimension *i* and there are finitely many. Otherwise, if $X_i$ is nonempty, it has dimension greater than *i* and *X* is not the union of finitely many $\mathfrak {g}$-leaves. In the case that $\mathrm{k} = \mathbf C$, there are finitely many analytic leaves of dimension *i* in an analytic neighborhood of every point if and only if $X_i$ has dimension *i* or is empty. See [25, Corollary 2.7] for more details.

The first corollary (and the original version of the result in [20]) is the following special case. Suppose that *X* is an affine Poisson variety, i.e., $\mathcal {O}(X)$ is equipped with a Lie bracket $\{-,-\}$ satisfying the Leibniz rule, $\{fg,h\}=f\{g,h\} + g\{f,h\}$ (called a Poisson bracket). Equivalently, $\mathcal {O}(X)$ is a Lie algebra such that the adjoint action, ${\text {ad}}(f):=\{f,-\}$, is by derivations. We use the notation $\xi _f := {\text {ad}}(f)$, which is called the Hamiltonian vector field of *f*. Let $\mathfrak {g}:= \mathcal {O}(X)$; then we have the action map $\alpha : \mathfrak {g}\rightarrow {\text {Vect}}(X)$ given by $\alpha (f) = \xi _f$. In this case, the $\mathfrak {g}$-leaves are called symplectic leaves, because for every $\mathfrak {g}$-leaf *Y* and every $y \in Y$, the tangent space $T_yY$ is equal to the space of restrictions $\xi _f|_y$ of all Hamiltonian vector fields $\xi _f$ at *y*. Then, it is easy (and standard) to see that the Lie bracket restricts to a well-defined Poisson bracket on each symplectic leaf, which is nondegenerate, i.e., it induces a symplectic structure determined by the formula $\omega (\xi _f) = df$.

In this case, the coinvariants $\mathcal {O}(X)_{\mathcal {O}(X)}$ are equal to $\mathcal {O}(X)/\{\mathcal {O}(X), \mathcal {O}(X)\}$, which is the zeroth Poisson homology of $\mathcal {O}(X)$ (and also the zeroth Lie homology of $\mathcal {O}(X)$ with coefficients in the adjoint representation $\mathcal {O}(X)$, i.e., the abelianization of $\mathcal {O}(X)$ as a Lie algebra). We denote it by ${\textsf {HP}}_0(\mathcal {O}(X))$.

### Corollary 2.6

[20, Theorem 3.1] Suppose that *X* is Poisson with finitely many symplectic leaves. Then ${\textsf {HP}}_0(\mathcal {O}(X)) = \mathcal {O}(X)/\{\mathcal {O}(X),\mathcal {O}(X)\}$ is finite-dimensional.

### Example 2.7

Suppose that *V* is a symplectic vector space and $G < \textsf {Sp}(V)$ is a finite subgroup. Then, as observed in [8, Sect. 7.4], the quotient $X = V/G := {\textsf {Spec}}\mathcal {O}(V)^G$ has finitely many symplectic leaves. These leaves can be explicitly described: call a subgroup $P < G$ *parabolic* if there exists $v \in V$ with stabilizer *P*. Let $X_P \subseteq X$ be the image of vectors in *V* whose stabilizer is conjugate to *P*. Then, $X_P$ is a symplectic leaf. To see this, let $v \in V$ have stabilizer *P*. Consider the projection $q: V \rightarrow V/G$. Then, the kernel of $dq|_v$ is precisely $(V^P)^\perp$. Hence, the differentials $d(q^* f)|_v$ for $f \in \mathcal {O}(V)^G$ form the dual space $\omega (V^P, -)$ to $V^P$ at *v*, which means that the Hamiltonian vector fields $\xi _{q^* f}$ restrict to $V^P$ at *v*. Since $dq|_{v}(V^P) = T_{q(v)} X_P$, we conclude that $T_{q(v)} X_P$ is indeed the space of restrictions of Hamiltonian vector fields $\xi _{f}$, as desired. To conclude that $X_P$ is a symplectic leaf, we have to show that it is connected. This follows since it is the image under a regular map of a connected set (an open subset of a vector space). As a result, we deduce the following corollary, which was a conjecture [1] of Alev and Farkas.

### Corollary 2.8

[5] If *V* is a symplectic vector space and $G < \textsf {Sp}(V)$ is a finite subgroup, then ${\textsf {HP}}_0(\mathcal {O}(V/G))=\mathcal {O}(V)^G/\{\mathcal {O}(V)^G,\mathcal {O}(V)^G\}$ is finite-dimensional.

In fact, the same result holds if *V* is not a symplectic vector space, but a symplectic affine variety, using the group $\textsf {SpAut}(V)$ of symplectic automorphisms of *V* (and $G < \textsf {SpAut}(V)$ still a finite subgroup). Again, we conclude that the symplectic leaves are the connected components of the $X_P$ as described above; the same proof applies except that the kernel of $dq|_v$ for $v \in V$ with stabilizer *P* is now $((T_v V)^P)^\perp$ (as we do not trivialize the bundle *TV*). Moreover, one has the following more general result:

### Corollary 2.9

[20, Corollary 1.3] If *V* is a symplectic vector space (or symplectic affine variety) and $G < \textsf {Sp}(V)$ (or $\textsf {SpAut}(V)$) is a finite subgroup, then $\mathcal {O}(V)/\{\mathcal {O}(V),\mathcal {O}(V)^G\}$ is finite-dimensional.

### Remark 2.10

For *V* a symplectic affine variety over $\mathbf k =\mathbf C$, we can give a more explicit formula for $\mathcal {O}(V)/\{\mathcal {O}(V),\mathcal {O}(V)^G\}$ [20, Corollary 4.20], which reduces it to the linear case and to some topological cohomology groups for local systems on locally closed subvarieties:

2.1 $$\begin{aligned} \mathcal {O}(V)/\{\mathcal {O}(V),\mathcal {O}(V)^G\} \cong \bigoplus _P \bigoplus _{Z \in C_P} H^{\dim Z}(Z, H(TV|_Z/TZ)), \end{aligned}$$

where *P* ranges over parabolic subgroups of *G* (stabilizers of points of *V*), $C_P$ is the set of connected components of $V^P$, and $H(TV|_Z/TZ)$ is the topological local system on *Z* whose fiber at $z \in Z$ is $\mathcal {O}(T_zV/T_zZ) / \{\mathcal {O}(T_zV/T_zZ),\mathcal {O}(T_zV,T_zZ)^P\}$, which carries a canonical flat connection by [20, Proposition 4.17] (induced along any path in *Z* from any choice of symplectic *P*-equivariant parallel transport along $T_zV/T_zZ$, and the choice will not matter on $H(TV|_Z/TZ)$ by definition).

### Remark 2.11

As observed in [20, Corollary 1.3], Corollary 2.9 continues to hold (with the same proof) if we only assume that *V* is an affine Poisson variety with finitely many leaves, and let *G* be a finite group acting by Poisson automorphisms.

### Example 2.12

One case of particular interest is that of symplectic resolutions. By definition, a resolution of singularities $\rho : {\widetilde{X}} \rightarrow X$ is a symplectic resolution if *X* is normal and ${\widetilde{X}}$ admits an algebraic symplectic form, i.e., a global nondegenerate closed two-form. Recall that a resolution of singularities is a proper, birational map such that ${\widetilde{X}}$ is smooth. In this situation, *X* is equipped with a canonical Poisson structure (fixing the symplectic form on ${\widetilde{X}}$): for every open affine subset $U \subseteq X$, one has $\mathcal {O}(U) = \Gamma (U, \mathcal {O}_X) = \Gamma (\rho ^{-1}(U), \mathcal {O}_{{\widetilde{X}}})$ since $\rho$ is proper and birational and *X* is normal. Thus, the Poisson structure on ${\widetilde{X}}$ gives $\mathcal {O}(U)$ a Poisson structure, which then gives $\mathcal {O}_X$ and hence *X* a Poisson structure. Conversely, if we begin with a normal Poisson variety *X*, we say that *X* admits a symplectic resolution if such a symplectic resolution ${\widetilde{X}} \rightarrow X$ exists, which recovers the Poisson structure on *X*.

Then, if *X* admits a symplectic resolution $\rho : {\widetilde{X}} \rightarrow X$, by [37, Theorem 2.5], it has finitely many symplectic leaves: indeed, for every closed irreducible subvariety $Y \subseteq X$ which is invariant under Hamiltonian flow, if $U \subseteq Y$ is the open dense subset such that the map $\rho |_{\rho ^{-1}(U)}: \rho ^{-1}(U) \rightarrow U$ is generically smooth on every fiber $\rho ^{-1}(u), u \in U$, then *U* is an open subset of a leaf. By induction on the dimension of *Y*, this shows that *Y* is a union of finitely many symplectic leaves; hence *X* is a union of finitely many symplectic leaves. See [37] for details.

We conclude that, in this case, ${\textsf {HP}}_0(\mathcal {O}(X))=\mathcal {O}(X)/\{\mathcal {O}(X),\mathcal {O}(X)\}$ is finite-dimensional.

### Example 2.13

More generally, a variety *X* is called a *symplectic singularity* [4] if it is normal, the smooth locus $X_{{\text {reg}}}$ carries a symplectic two-form $\omega _{{\text {reg}}}$, and for any resolution $\rho : \widetilde{X} \rightarrow X$, $\rho |_{\rho ^{-1}(X_{{\text {reg}}})}^* \omega _{{\text {reg}}}$ extends to a regular (but not necessarily nondegenerate) two-form on *X*. (This condition is independent of the choice of resolution, as explained in [4], since a rational differential form an a smooth variety is regular if and only if its pullback under a proper birational map is regular.) By [37, Theorem 2.5], every symplectic singularity has finitely many symplectic leaves. Therefore, ${\textsf {HP}}_0(\mathcal {O}(X))$ is finite-dimensional.

### Remark 2.14

By definition, every variety admitting a symplectic resolution is a symplectic singularity. However, the converse is far from true. Let $\mathbf k = \mathbf C$. By [4, Proposition 2.4], any quotient of a symplectic singularity by a finite group preserving the generic symplectic form is still a symplectic singularity. But even for a symplectic vector space *V* it is far from true that *V* / *G* admits a symplectic resolution for all $G < \textsf {Sp}(V)$ finite. To admit a resolution, by Verbitsky’s theorem [58], *G* must be generated by symplectic reflections (elements $g \in G$ with $\ker (g-{\text {Id}}) \subseteq V$ having codimension two). Moreover, a series of works [6, 11, 12, 15, 29] leads to the expectation that every quotient *V* / *G* admitting a symplectic resolution is a product of factors of the form $\mathbf C ^{2n}/(\Gamma ^n \rtimes S_n)$ for $\Gamma < \textsf {SL}(2,\mathbf C )$ finite, or of two exceptional factors of dimension four (by [6, 15, 29], this holds at least when *G* preserves a Lagrangian subspace $U \subseteq V$ and hence can be viewed as a subgroup of $\textsf {GL}(U)$, and in general by [12] there is a list of cases of groups in dimension $\le 10$ which remain to check).

### Example 2.15

Let *V* be a symplectic vector space and $G<\textsf {Sp}(V)$ a reductive subgroup. There is a natural moment map, $\mu : V \rightarrow \mathfrak {g}^*$ with $\mathfrak {g}= {\textsf {Lie}}\, G$, defined as follows. Let $\mathfrak {sp}(V) = {\textsf {Lie}}\textsf {Sp}(V)$, and note $\mathfrak {sp}(V) \cong {\textsf {Sym}}^2 V^*$ with the Poisson structure on ${\textsf {Sym}}V^* \cong \mathcal {O}(V)$ induced by the symplectic form. Then, the moment map $V \rightarrow \mathfrak {sp}(V)^* \cong {\textsf {Sym}}^2 V$ is the squaring map, $v \mapsto v^2$, and by restriction we get a moment map $\mu : V \rightarrow \mathfrak {g}^*$. We can then define the Hamiltonian reduction, $X:= \mu ^{-1}(0)//G := {\textsf {Spec}}\, \mathcal {O}(\mu ^{-1}(0))^G$. This is well known to inherit a Poisson structure, which on functions is given by the same formula as that for the Poisson bracket on $\mathcal {O}(V)$. In general, *X* need not be reduced, but by [46, Sect. 2.3], the reduced subscheme $X^{{\text {red}}}$ has finitely many symplectic leaves. These leaves are explicitly given as the irreducible components of the locally closed subsets $X^{{\text {red}}}_P = \{q(x) \mid x \in \mu ^{-1}(0), G_x=P, G \cdot x \text { is closed}\} \subseteq X^{{\text {red}}}$, where $q: \mu ^{-1}(0) \rightarrow X$ is the quotient map. Therefore, ${\textsf {HP}}_0(\mathcal {O}(V/G))$ is finite-dimensional (since Theorem 2.4 also applies to Poisson schemes that need not be reduced). Note that this example subsumes Example 2.7 (which is the special case where *G* is finite).

## Irreducible representations of quantizations

**3.1.** We now apply the preceding results to the study of quantizations of Poisson varieties. Let *X* be an affine Poisson variety. Recall the following standard definitions:

### Definition 3.1

A deformation quantization of *X* is an associative algebra $A_\hbar$ over $\mathbf k \llbracket \hbar \rrbracket$ of the form $A_\hbar = (\mathcal {O}(X)\llbracket \hbar \rrbracket , \star )$ where $\mathcal {O}(X) \llbracket \hbar \rrbracket := \{\sum _{m \ge 0} a_m \hbar ^m \mid a_m \in \mathcal {O}(X)\}$, and $\star$ is an associative $\mathbf k \llbracket \hbar \rrbracket$-linear multiplication such that $a \star b \equiv ab \pmod \hbar$ and $a \star b - b \star a \equiv \hbar \{a,b\} \pmod {\hbar ^2}$ for all $a,b \in \mathcal {O}(X)$.

### Remark 3.2

Note that the multiplication $\star$ is automatically continuous in the $\hbar$-adic topology, since $(\hbar ^m A_\hbar ) \star (\hbar ^n A_\hbar ) \subseteq \hbar ^{m+n} A_\hbar$.

### Definition 3.3

If $\mathcal {O}(X)$ is nonnegatively graded with Poisson bracket of degree $-d < 0$, then a filtered quantization is a filtered associative algebra $A = \bigcup _{m \ge 0} A_{\le m}$ such that ${\textsf {gr}}A := \bigoplus _{m \ge 0} A_{\le m} / A_{\le m-1} \cong \mathcal {O}(X)$ and such that, for $a \in A_{\le m}, b \in A_{\le n}$, then $ab-ba \in A_{\le m+n-d}$ and ${\textsf {gr}}_{m+n-d} (ab-ba) = \{{\textsf {gr}}_m a,{\textsf {gr}}_n b\}$.

Given a deformation quantization $A_\hbar$, we consider the $\mathbf k ((\hbar ))$-algebra $A_\hbar [\hbar ^{-1}]$.

### Theorem 3.4

Assume that *X* is an affine Poisson variety with finitely many symplectic leaves. Then, for every deformation quantization $A_\hbar$, there are only finitely many continuous irreducible finite-dimensional representations of $A_\hbar [\hbar ^{-1}]$. If $\mathcal {O}(X)$ is nonnegatively graded with Poisson bracket of degree $-d < 0$ and *A* is a filtered quantization, then there are only finitely many irreducible finite-dimensional representations of *A* (over $\mathbf k$).

Here by continuous we mean that the map $\rho : A_\hbar [\hbar ^{-1}] \rightarrow \text {Mat}_n(\mathbf k ((\hbar )))$ is continuous in the $\hbar$-adic topology, i.e., for some $m \in \mathbf Z$, we have $\rho (A_\hbar ) \subseteq \hbar ^m \text {Mat}_n(\mathbf k \llbracket \hbar \rrbracket )$. The basic tool we use is a standard result from Wedderburn theory:

### Proposition 3.5

If *A* is an algebra over a field *F*, then the characters (i.e., traces) of nonisomorphic irreducible finite-dimensional representations of *A* over *F* are linearly independent over *F*.

### Proof of Theorem 3.4

We begin with the second statement. Note that [*A*, *A*] is a filtered subspace of *A*, and hence, $\textsf {HH}_0(A)=A/[A,A]$ is also filtered. By definition we have $\{\mathcal {O}(X), \mathcal {O}(X) \} \subseteq {\textsf {gr}}[A,A]$. Therefore, we obtain a surjection ${\textsf {HP}}_0(\mathcal {O}(X))=\mathcal {O}(X)/\{\mathcal {O}(X),\mathcal {O}(X)\} \twoheadrightarrow {\textsf {gr}}\, \textsf {HH}_0(A)={\textsf {gr}}\, A/[A,A]$. As a result, $\dim \textsf {HH}_0(A) =\dim {\textsf {gr}}\textsf {HH}_0(A) \le \dim {\textsf {HP}}_0(\mathcal {O}(X))$, which is finite by Theorem 2.4.

Given a finite-dimensional representation $\rho :A \rightarrow {\text {End}}(V)$ of *A*, the character $\chi _\rho := {\text {tr}} \chi$ is a linear functional $\chi \in A^*$. As traces annihilate commutators, $\chi _\rho \in \textsf {HH}_0(A)^* = [A,A]^\perp \subseteq A^*$. By Proposition 3.5, we conclude that the number of such representations cannot exceed $\dim \textsf {HH}_0(A)^*$. By the preceding paragraph, this is finite dimensional, so there can only be finitely many irreducible finite-dimensional representations of *A* (at most $\dim {\textsf {HP}}_0(\mathcal {O}(X))$).

For the first statement, the same proof applies, except that now we need to take some care with the $\hbar$-adic topology. Namely, let $\overline{[A_\hbar ,A_\hbar ]}$ be the closure of $[A_\hbar ,A_\hbar ]$ in the $\hbar$-adic topology, i.e., $\{\sum _{m \ge 0} \hbar ^m c_m \mid c_m \in [A_\hbar ,A_\hbar ]\}$. Let $V \subseteq \mathcal {O}(X)$ be a finite-dimensional subspace such that the composition $V \hookrightarrow \mathcal {O}(X) \twoheadrightarrow {\textsf {HP}}_0(\mathcal {O}(X))$ is an isomorphism. We claim that $V \llbracket \hbar \rrbracket \hookrightarrow A_\hbar \twoheadrightarrow A_\hbar /\hbar ^{-1}\overline{[A_\hbar ,A_\hbar ]}$ is a surjection. This follows from the following lemma:

### Lemma 3.6

$$A_\hbar \subseteq V \llbracket \hbar \rrbracket + \hbar ^{-1}\overline{[A_\hbar ,A_\hbar ]}$$.

### Proof

We claim that, for every $m \ge 1$, $A_\hbar \subseteq V \llbracket \hbar \rrbracket + \hbar ^{-1}\overline{[A_\hbar ,A_\hbar ]} + \hbar ^m A_\hbar$. We prove it by induction on *m*. For $m=1$ this is true by definition of *V*. Therefore also $\hbar A_\hbar \subseteq V \llbracket \hbar \rrbracket + \hbar ^{-1}\overline{[A_\hbar ,A_\hbar ]} + \hbar ^2 A_\hbar$. For the inductive step, if $A_\hbar \subseteq V \llbracket \hbar \rrbracket + \hbar ^{-1}\overline{[A_\hbar ,A_\hbar ]} + \hbar ^m A_\hbar$ for $m \ge 1$, then substituting the previous equation into $\hbar ^m A_\hbar = \hbar ^{m-1} (\hbar A_\hbar )$, we obtain the desired result.

Since $V \llbracket \hbar \rrbracket$ and $\overline{[A_\hbar ,A_\hbar ]}$ are closed subspaces of $A_\hbar$, it follows that $A_\hbar \subseteq V \llbracket \hbar \rrbracket + \hbar ^{-1} \overline{[A_\hbar ,A_\hbar ]}$ which proves the lemma. $\square$

Next, let $d = \dim {\textsf {HP}}_0(\mathcal {O}(X))$ and suppose that $\chi _1, \ldots , \chi _{d+1}$ are characters of nonisomorphic continuous irreducible representations of $A_\hbar [\hbar ^{-1}]$ over $\mathbf k ((\hbar ))$. Then, there exist $a_1, \ldots , a_{d+1} \in \mathbf k \llbracket \hbar \rrbracket$, not all zero, such that $\sum _i a_i \chi _i|_{V \llbracket \hbar \rrbracket } = 0$, since $\dim V = d < d+1$. Since the representations were continuous, $\chi _i(\hbar ^{-1}\overline{[A_\hbar ,A_\hbar ]})=0$ for all *i*. By Lemma 3.6, $\sum _i a_i \chi _i|_{A_\hbar }=0$, and by $\mathbf k ((\hbar ))$-linearity, $\sum _i a_i \chi _i = 0$. This again contradicts Proposition 3.5. $\square$

### Remark 3.7

The proof actually implies the stronger result that $A_\hbar \otimes _\mathbf{k \llbracket \hbar \rrbracket } K$ has finitely many continuous irreducible finite-dimensional representations over *K* (also at most $\dim {\textsf {HP}}_0(\mathcal {O}(X))$), where $K = \overline{\mathbf{k ((\hbar ))}}$ is the algebraic closure of $\mathbf k ((\hbar ))$ (the field of Puiseux series over $\mathbf k$, i.e., $\bigcup _{r \ge 1} \mathbf k ((\hbar ^{1/r}))$). Here an *n*-dimensional representation $\rho : A_\hbar \otimes _\mathbf{k \llbracket \hbar \rrbracket } K \rightarrow \text {Mat}_n(K)$ is continuous if $\rho (A_\hbar ) \subseteq \hbar ^m \text {Mat}_n(\mathcal {O}_K)$ for some $m \in \mathbf Z$, where $\mathcal {O}_K=\bigcup _{r \ge 1} \mathbf k \llbracket \hbar ^{1/r}\rrbracket$ is the ring of integers of *K*. This result is stronger since if $\rho _1, \rho _2$ are two nonisomorphic irreducible representations of an algebra *A* over a field *F*, then for any extension field *E*, ${\text {Hom}}_{A \otimes _F E} (\rho _1 \otimes _F E, \rho _2 \otimes _F E) = {\text {Hom}}_A(\rho _1, \rho _2) \otimes _F E = 0$, so all irreducible representations occurring over *E* in $\rho _1 \otimes _F E$ and $\rho _2 \otimes _F E$ are distinct.

## Proof of Theorem 2.4 using D-modules

In this section, we explain the proof of Theorem 2.4. We need the theory of holonomic D-modules. (The necessary definitions and results are recalled in the appendix; see, e.g., [36] for more details.) An advantage of using D-modules is that the approach is local and hence does not essentially require affine varieties. However, for simplicity (to avoid, for example, presheaves of Lie algebras of vector fields), we will explain the theory for affine varieties and then indicate how it generalizes.

### The affine case

The main idea is the following construction. Given an affine Poisson variety *X* and a Lie algebra $\mathfrak {g}$ acting on *X* via a map $\alpha : \mathfrak {g}\rightarrow {\text {Vect}}(X)$, we construct a D-module $M(X,\mathfrak {g})$ which represents the functor of invariants under the flow of $\mathfrak {g}$, i.e., such that ${\text {Hom}}(M(X,\mathfrak {g}), N) = N^{\mathfrak {g}}$ for all D-modules *N*, where we will define $N^{\mathfrak {g}}$ below. Without loss of generality, let us assume $\mathfrak {g}\subseteq {\text {Vect}}(X)$ and that $\alpha$ is the inclusion; otherwise, we replace $\mathfrak {g}$ by its image $\alpha (\mathfrak {g})$. Let $i: X \rightarrow V$ be any closed embedding into a smooth affine variety *V*. Denote the ideal of *i*(*X*) in *V* by $I_X$. Let ${\widetilde{\mathfrak {g}}} \subseteq {\text {Vect}}(V)$ be the Lie subalgebra of vector fields which are tangent to *X* and whose restriction to *X* is in the image of $\alpha$. As recalled in Sect. A.1, there are mutually quasi-inverse functors $i_{\natural }: \mathcal {D}-\text {mod}_X\rightarrow \text {mod}_X-\mathcal {D}(V)$ and $i^{\natural }: \text {mod}_X-\mathcal {D}(V) \rightarrow \mathcal {D}-\text {mod}_X$ defined in Sect. A.1, where $\text {mod}_X-\mathcal {D}(V)$ denotes the category of *right* modules over $\mathcal {D}(V)$ supported on *i*(*X*), and $\mathcal {D}-\text {mod}_X$ is the category of D-modules on *X*; this is in fact the way we define the category $\mathcal {D}-\text {mod}_X$. (We will call these merely D-modules on *X*, since using left D-modules gives an equivalent definition: see Remark A.10.)

#### Definition 4.1

[20, Definition 2.2], [25, 2.12] $$M(X,\mathfrak {g}) := i^{\natural } \bigl ( ({\widetilde{\mathfrak {g}}} \cdot \mathcal {D}(V) + I_X \cdot \mathcal {D}(V)) {\setminus } \mathcal {D}(V) \bigr )$$.

We will often work with $\mathcal {O}(V)$-coherent right $\mathcal {D}(V)$-modules supported on *i*(*X*). Note that, on a smooth variety, such modules are well known to be vector bundles on *X*. (In more detail, one composes the equivalence between right and left D-modules on smooth varieties with the equivalence between $\mathcal {O}$-coherent left D-modules on a smooth variety and vector bundles with flat algebraic connections.)

#### Example 4.2

Suppose that $\mathfrak {g}$ acts transitively on *X*; in particular, this means *X* is smooth, so we can take $V=X$. Assume also that *X* is connected. In this case, by [25, Proposition 2.36], $M(X,\mathfrak {g})$ is either a line bundle or zero. (This can be shown by a straightforward computation of its associated graded module over $\mathcal {O}(T^*X)$, cf. the proof of Lemma 4.6 below.) In the case that $\mathfrak {g}$ preserves a global nonvanishing volume form (which is sometimes called an affine Calabi–Yau structure), we obtain $\Omega _X$, the canonical right $\mathcal {D}_X$-module of volume forms; the isomorphism $M(X,\mathfrak {g}) \rightarrow \Omega _X$ sends the image of $1 \in \mathcal {D}(X) \twoheadrightarrow M(X,\mathfrak {g})$ to the nonvanishing volume form. This includes the situation where *X* is symplectic and $\mathfrak {g}$ is either $\mathcal {O}(X)$ or its image in ${\text {Vect}}(X)$, the Lie algebra of Hamiltonian vector fields on *X*.

Given any D-module *N* on *X*, let $\Gamma _{\mathcal {D}}(X,N) := {\text {Hom}}_{\mathcal {D}(V)}(\mathcal {D}_X, i_\natural N)$ be the sections of *N* supported on *i*(*X*) (see Sect. A.2 for more details). For $\xi \in \mathfrak {g}$, and any lift ${\widetilde{\xi }} \in {\widetilde{\mathfrak {g}}}$, we have a linear endomorphism of $\Gamma (V,i^{\natural } N)$ given by right multiplication by ${\widetilde{\xi }}$. This preserves the linear subspace $\Gamma _{\mathcal {D}}(X,N)$. The resulting endomorphism does not depend on the choice of the lift $\widetilde{\xi }$ and defines a Lie algebra action of $\mathfrak {g}$ on $\Gamma _{\mathcal {D}}(X,N)$. Therefore, we may consider the vector space $N^\mathfrak {g}:= H^0(\mathfrak {g}, \Gamma _{\mathcal {D}}(X,N)) = \{n \in \Gamma _{\mathcal {D}}(X,N) \mid n \cdot \xi = 0, \forall \xi \in \mathfrak {g}\}$.

#### Lemma 4.3

Definition 4.1 does not depend on the choice of closed embedding $X \rightarrow V$. Moreover, for every D-module *N* on *X*, we have ${\text {Hom}}(M(X,\mathfrak {g}), N) = N^\mathfrak {g}$.

The purpose of the second statement above is to explain what functor is represented by $M(X,\mathfrak {g})$.

#### Proof

The first statement follows from the following alternative definition of $M(X,\mathfrak {g})$. Note that $\mathfrak {g}$ acts on the D-module $\mathcal {D}_X$ (see Sect. A.2 for its definition) on the left by D-module endomorphisms: there is a canonical generator $1 \in \mathcal {D}_X$, so for any $\xi \in \mathfrak {g}$ and lift ${\widetilde{\xi }} \in {\widetilde{\mathfrak {g}}}$, we can define $\xi \cdot 1 = {\widetilde{\xi }} \in i_{\natural }\mathcal {D}_X = I_X \cdot \mathcal {D}_V {\setminus } \mathcal {D}_V$, and this does not depend on the choice of ${\widetilde{\xi }}$. This extends uniquely to the claimed action. Then, one may check that $M(X,\mathfrak {g}) = \mathfrak {g}\cdot \mathcal {D}_X {\setminus } \mathcal {D}_X$. From this one easily deduces the second statement. $\square$

#### Remark 4.4

We see from the proof that there is a canonical surjection $\mathcal {D}_X \rightarrow M(X,\mathfrak {g})$. Equivalently, there is a canonical global section $1 = 1_{M(X,\mathfrak {g})} \in \Gamma _{\mathcal {D}}(X,M(X,\mathfrak {g})) = {\text {Hom}}(\mathcal {D}_X, M(X,\mathfrak {g}))$. For every closed embedding $i: X \rightarrow V$ into a smooth affine variety, applying $i_\natural$ to this map and taking the composition $\mathcal {D}(V) \twoheadrightarrow i_\natural \mathcal {D}_X \twoheadrightarrow i_\natural M(X,\mathfrak {g})$, we get a canonical generator $1 \in i_\natural M(X,\mathfrak {g})$ as a right module over $\mathcal {D}(V)$. This is nothing but the image of $1 \in \mathcal {D}(V)$ under the defining surjection $\mathcal {D}(V) \rightarrow i_\natural M(X,\mathfrak {g})$. We will make use of this canonical generator below.

Let $\pi : X \rightarrow \text {pt}$ be the projection to a point. We will need the functor of underived direct image, $H^0\pi _{*}$ (see the appendix for the definition). Then, we have the following fundamental relationship between the pushforward to a point of $M(X,\mathfrak {g})$ and coinvariants of $\mathcal {O}(X)$.

#### Lemma 4.5

$$H^0\pi _{*} M(X,\mathfrak {g}) \cong \mathcal {O}(X)_\mathfrak {g}$$.

#### Proof

Recall from (A.1) and (A.3) that

4.1 $$\begin{aligned} H^0\pi _{*} M(X,\mathfrak {g})= & {} (i_{\natural } M(X,\mathfrak {g})) \otimes _{\mathcal {D}(V)} \mathcal {O}(V) = ({\widetilde{\mathfrak {g}}} \cdot \mathcal {D}_V + I_X \cdot \mathcal {D}_V) {\setminus } \mathcal {D}_V \otimes _{\mathcal {D}_V} \mathcal {O}_V \nonumber \\\cong & {} \mathcal {O}_V / ({\widetilde{\mathfrak {g}}} \cdot \mathcal {O}_V + I_X) \cong \mathcal {O}_X / (\mathfrak {g}\cdot \mathcal {O}_X) = (\mathcal {O}_X)_\mathfrak {g}. \end{aligned}$$

$$\square $$

The proof of Theorem 2.4 rests on an estimate for the characteristic variety (singular support) of $M(X,\mathfrak {g})$ (whose definition we recall in Definition A.13). Recall above that a $\mathfrak {g}$-leaf is smooth. Therefore, given a closed embedding $X \rightarrow V$ into a smooth variety, each $\mathfrak {g}$-leaf *Z* has a well-defined conormal bundle, which we denote by $T_Z^* V$, which has dimension equal to the dimension of *V*.

#### Lemma 4.6

Suppose *X* is the union of finitely many $\mathfrak {g}$-leaves and $i: X \rightarrow V$ is a closed embedding into a smooth variety. Then, the characteristic variety of $i_\natural M(X,\mathfrak {g})$ is contained in the union of the conormal bundles of these $\mathfrak {g}$-leaves inside *V*.

#### Proof

We make an explicit (and straightforward) computation. For notational convenience, we assume that $X \subseteq V$ and that *i* is the inclusion. We equip $i_\natural M(X,\mathfrak {g})$ with the good filtration given by the canonical generator $1 \in i_\natural M(X,\mathfrak {g})$, i.e., $\bigl ( i_\natural M(X,\mathfrak {g}) \bigr )_{\le m} = (\mathcal {D}_V)_{\le m} \cdot 1$; see Remark 4.4. Then, we claim that the associated graded relations of the defining relations, $I_X$ and ${\widetilde{\mathfrak {g}}}$, of $i_\natural M(X,\mathfrak {g})$, cut out the union of the conormal bundles. In this case the associated graded relations are also just $I_X, \widetilde{\mathfrak {g}} \subseteq \mathcal {O}(T^*V)$. Then, in view of the canonical surjection $\mathcal {O}(T^* V)/(I_X \mathcal {O}(T^* V) + {\widetilde{\mathfrak {g}}} \mathcal {O}(T^* V)) \twoheadrightarrow {\textsf {gr}}\, i_\natural M(X,\mathfrak {g})$, we obtain the result.

The claim follows from a more general one that does not require the assumption that *X* is the union of finitely many $\mathfrak {g}$-leaves:

$$\begin{aligned} Z(I_X + {\widetilde{\mathfrak {g}}}) = \{(x, p): x \in X,\,\, p \in {\text {im}}(\alpha _x)^\perp \}. \end{aligned}$$

By definition, the restriction of the RHS to any $\mathfrak {g}$-leaf is the conormal bundle to the leaf, which proves the preceding claim. To prove the above formula, note first that $I_X \mathcal {O}(T^*V)$ is nothing but the ideal $I_{T^* V|_X}$ of the subset $T^* V|_X$, the restriction of the cotangent bundle to $X \subseteq V$. Then, at each $x \in X$, the equations ${\widetilde{\mathfrak {g}}}$ cut out, in the cotangent fiber $T^*_x V$, the perpendicular ${\text {im}}(\alpha _x)^\perp$. This proves the claim, and hence the lemma. $\square$

Since the conormal bundle to a smooth subvariety $Z \subseteq V$ of a smooth variety *V* has dimension equal to the dimension of *V*, we conclude:

#### Theorem 4.7

If *X* is the union of finitely many $\mathfrak {g}$-leaves, then $M(X,\mathfrak {g})$ is holonomic.

We remark that this result, in the case that $\mathfrak {g}$ is the derivative of the action of a (connected) algebraic group *G* on *X*, is well known (see, e.g., [35, Section 5]); see also Remark 6.10 below. We can now complete the proof of Theorem 2.4. By Theorem 4.7 and Corollary A.19, $H^0\pi _{*} M(X,\mathfrak {g})$ is finite dimensional. By Lemma 4.5, this is $\mathcal {O}(X)_\mathfrak {g}$, which is hence finite dimensional.

#### Example 4.8

Suppose that $X=V/G$ for *V* a symplectic vector space and $G < \textsf {Sp}(V)$ a finite group of symplectic automorphisms. For any parabolic subgroup *P* in *G* (as in Example 2.7), let *N*(*P*) be the normalizer of *P* in *G*, and $N^0(P) := N(P)/P$. Let $i_K: V^P/N^0(P)\rightarrow V/G$ be the corresponding closed embedding. Then by [20, Corollary 4.16], there is a canonical isomorphism, where $\text {Par}(G)/G$ denotes the set of conjugacy classes [*P*] of parabolic subgroups $P < G$,

4.2 $$\begin{aligned} M(V/G) \cong \bigoplus _{[P] \in \text {Par}(G)/G} {\textsf {HP}}_0\left( \mathcal {O}_{(V^P)^\perp /P}\right) \otimes (i_K)_*\left( {\text {IC}}\left( V^P/N^0(P)\right) \right) . \end{aligned}$$

### Globalization

In this subsection, we briefly explain how to generalize the previous constructions to the not necessarily affine case. If *X* is an arbitrary variety, then we may consider a presheaf $\mathfrak {g}$ of Lie algebras acting on *X* via a map $\alpha : \mathfrak {g}\rightarrow T_X$. For example, $\mathfrak {g}$ could be a constant sheaf, giving a (global) action of $\mathfrak {g}$ on *X*. Another example is if *X* is a Poisson variety; then $\mathfrak {g}$ could be $\mathcal {O}_X$, acting by the Poisson bracket, or its image in $T_X$, which is the presheaf of Hamiltonian vector fields.

As before, without loss of generality, let us assume that $\mathfrak {g}\subseteq T_X$ is a sub-presheaf and $\alpha$ is the inclusion (we can just take the image of $\alpha$). Let $i: X \rightarrow V$ be a closed embedding into a smooth variety *V* and $\mathcal {I}_X$ the ideal sheaf of *i*(*X*). Let ${\widetilde{\mathfrak {g}}} \subseteq T_V$ be the sub-presheaf of vector fields which are tangent to *X* and restrict on *X* to vector fields in $\mathfrak {g}$. Then, given any open affine subset $U \subseteq X$, we can consider the D-module $M(U,\mathfrak {g}(U))$ defined as in Definition 4.1. Under mild conditions, these then glue together to form a D-module on *X*:

#### Definition 4.9

[25, Definition 3.4] The presheaf $\mathfrak {g}$ is $\mathcal {D}$ *-localizable* if, for every chain $U' \subseteq U \subseteq X$ of open affine subsets,

4.3 $$\begin{aligned} \mathfrak {g}(U') \cdot \mathcal {D}(U') = \mathfrak {g}(U) \cdot \mathcal {D}(U'). \end{aligned}$$

In particular, it is immediate that if $\mathfrak {g}$ is a constant sheaf, then it is $\mathcal {D}$-localizable. By [25, Example 3.11], the presheaf of Hamiltonian vector fields on an arbitrary Poisson variety is $\mathcal {D}$-localizable. By [25, Example 3.9], the sheaf of all vector fields is $\mathcal {D}$-localizable (note that merely being a sheaf does not imply $\mathcal {D}$-localizability, although by [25, Example 3.10], being a quasi-coherent sheaf acting in a certain way does imply $\mathcal {D}$-localizability).

#### Proposition 4.10

[25, Proposition 3.5] If $\mathfrak {g}$ is $\mathcal {D}$-localizable, then there is a canonical D-module $M(X,\mathfrak {g})$ on *X* whose restriction to every open affine *U* is $M(U,\mathfrak {g}(U))$.

#### Remark 4.11

In [25], the above is stated somewhat more generally: one can fix an open affine covering and ask only that $\mathfrak {g}$ be $\mathcal {D}$-localizable with respect to this covering. Above we take the covering given by *all* open affine subsets, and it turns out that the $\mathfrak {g}$ we are interested in are all $\mathcal {D}$-localizable with respect to that covering (which is the strongest $\mathcal {D}$-localizability condition).

With this definition, all of the results and proofs of the previous section, except for Lemma 4.5 (and the proof of Theorem 2.4) carry over. In particular, Theorem 4.7 holds for arbitrary (not necessarily affine) *X*.

#### Example 4.12

As in Example 4.2, we can consider the case where $\mathfrak {g}$ acts transitively on *X*. Assume *X* is irreducible. As before, *X* is smooth, so $M(X,\mathfrak {g})$ is either a line bundle or zero. If $\mathfrak {g}$ preserves a nonvanishing global volume form, we again deduce that $M(X,\mathfrak {g}) = \Omega _X$, by the same isomorphism sending $1 \in M(X,\mathfrak {g})$ to the volume form. This includes the case that *X* is symplectic (and need not be affine), with the symplectic volume form.

#### Corollary 4.13

If *X* is a (not necessarily affine) Poisson variety with finitely many symplectic leaves, and $\mathfrak {g}$ is $\mathcal {O}_X$ (or the presheaf of Hamiltonian vector fields), then $M(X,\mathfrak {g})$ is holonomic.

#### Proof

We only need to observe that the $\mathfrak {g}$-leaves are the symplectic leaves. Then, the result follows from Theorem 4.7.

#### Remark 4.14

Beware that the use of presheaves above is necessary: for a general Poisson variety *X*, the presheaf $\mathfrak {g}$ of Hamiltonian vector fields is *not* a sheaf: see [25, Remark 3.16] (even though $\mathfrak {g}$ is the image of the action $\alpha : \mathcal {O}_X \rightarrow T_X$ of the honest sheaf of Lie algebras $\mathcal {O}_X$ on *X*). However, as we observed there, when *X* is generically symplectic (which is true when it has finitely many symplectic leaves, as in all of our main examples), then $\mathfrak {g}$ is a sheaf (although it is clearly not quasi-coherent).

#### Example 4.15

Suppose that *V* is a symplectic variety (not necessarily affine) and $G < \textsf {SpAut}(V)$ a finite subgroup of symplectic automorphisms. Then we can let $\mathfrak {g}:= H(V)^G$, the *G*-invariant Hamiltonian vector fields. Then, we obtain the following formula [20, Theorem 4.19], which implies (2.1) from before. In the notation of Remark 2.10, for $i_Z: Z \rightarrow V$ the closed embedding:

4.4 $$\begin{aligned} M(V,\mathfrak {g}) \cong \bigoplus _P \bigoplus _{Z \in C_P} (i_Z)_* H(TV|_Z/TZ), \end{aligned}$$

where the sum is over all parabolic subgroups of *V*, and we view topological local systems on smooth subvarieties of *V* as left D-modules and hence right D-modules in the canonical way.

Passing to *V* / *G* we get a global generalization of Example 4.8 [20, Theorem 4.21]: let $\text {Par}(G)/G$ be the set of conjugacy classes [*P*] of parabolic subgroups $P < G$, and for $Z \in C_P$, let $N_Z(P) < N(P)$ be the subgroup of elements of the normalizer *N*(*P*) of *P* which map *Z* to itself, and let $N_Z^0(P) := N_Z(P)/P$. Let $Z_0 := Z/N^0_Z(K)$ and $i_{Z_0}: Z_0 \rightarrow V/G$ the closed embedding. Let $\pi _Z: Z \rightarrow Z_0$ be the $N_Z^0(P)$-covering, and let $H_{Z_0} := (\pi _Z)_* H(TV_Z/TZ)^{N_Z^0(P)}$ be the D-module on $Z_0$ obtained by equivariant pushforward from *Z*. Then we obtain:

4.5 $$\begin{aligned} M(V/G) \cong \bigoplus _{[P] \in \text {Par}(G)/G} \bigoplus _{Z \in C_P/N(P)} (i_{Z_0})_* H_{Z_0}. \end{aligned}$$

#### Remark 4.16

In fact, one can prove a $C^\infty$ analogue of (4.4), using the analogous (but simpler) arguments for distributions rather than D-modules. Let *V* be a compact $C^\infty$-manifold and *G* be a finite group acting faithfully on *V*. For $P \le G$ a parabolic subgroup and *Z* a connected component of the locus of fixed points of *P*, let $H_Z$ denote the space of flat sections of the local system $H(TV|_Z/TZ)$ recalled in Remark 2.10: by [20, Sect. 4.6], the local system is trivial, so $H_Z$ identifies with each fiber: $H_Z \cong \mathcal {O}(T_zV/T_zZ) / \{\mathcal {O}(T_zV/T_zZ),\mathcal {O}(T_zV,T_zZ)^P\}$ for every $z \in Z$. In particular, by Corollary 2.9, $H_Z$ is a finite-dimensional vector space. Then, [20, Proposition 4.23] states that the space of smooth distributions on *V* invariant under *G*-invariant Hamiltonian vector fields is isomorphic to

4.6 $$\begin{aligned} \bigoplus _P \bigoplus _{Z \in C_P} H_Z^*. \end{aligned}$$

In particular, it is finite dimensional and of dimension $\sum _P \sum _{Z \in C_P} \dim H_Z$.

#### Remark 4.17

Theorem 4.7 continues to hold in the complex analytic setting, using the results of this section. However, the coinvariants $\mathcal {O}(X)_\mathfrak {g}$ need not be finite dimensional: for instance, if $X = \mathbf C ^\times \times (\mathbf C {\setminus } \mathbf Z )$ equipped with the usual symplectic form from the inclusion $X \subseteq \mathbf C ^2$ and $\mathfrak {g}=\mathcal {O}(X)$, then $\mathcal {O}(X)_\mathfrak {g}\cong H^2(X)$, which is infinite-dimensional.

## Poisson-De Rham and $\mathfrak {g}$-de Rham homology

**5.1.** As an application of the constructions of the previous section, we can define a new derived version of the coinvariants $\mathcal {O}(X)_\mathfrak {g}$. Let *X* be an affine variety and $\mathfrak {g}$ a Lie algebra acting on *X*.

### Definition 5.1

The $\mathfrak {g}$-de Rham homology of *X*, $H^{\mathfrak {g}-DR}_*(X)$, is defined as the full derived pushforward $H^{\mathfrak {g}-DR}_i(X) := H^{-i}(\pi _* M(X,\mathfrak {g}))$.

By Lemma 4.5, $H^{\mathfrak {g}-DR}_0(X)=\mathcal {O}(X)_\mathfrak {g}$. In this case, the pushforward functor $H^0\pi _*$ is right exact, and $H^{-i}(\pi _*) = L^i (H^0\pi _*)$ is the *i*-th left derived functor (which is why we negate the index *i* and define a homology theory, rather than a cohomology theory).

Using Sect. 4.2, this definition carries over to the nonaffine setting, where now $\mathfrak {g}$ may be an arbitrary $\mathcal {D}$-localizable presheaf of vector fields on *X*; however, we no longer have an (obvious) interpretation of $H^{\mathfrak {g}-DR}_0(X)$ (and $H^0\pi _*$ is no longer right exact in general).

### Example 5.2

In the case that *X* is Poisson, Definition 5.1 defines a homology theory which we call the *Poisson-de Rham homology*. Recall here that, when *X* is affine, we let $\mathfrak {g}$ be $\mathcal {O}(X)$ (or its image in ${\text {Vect}}(X)$, the Lie algebra of Hamiltonian vector fields on *X*). For general *X*, we let $\mathfrak {g}$ be $\mathcal {O}_X$ (or its image in $T_X$, the presheaf of Lie algebras of Hamiltonian vector fields). We denote this theory by ${\textsf {HP}}^{DR}_*(X) = H^{\mathfrak {g}-DR}_*(X)$. If *X* is affine, then ${\textsf {HP}}^{DR}_0(X) = {\textsf {HP}}_0(\mathcal {O}(X))$ is the (ordinary) zeroth Poisson homology. If *X* is symplectic, then we claim that ${\textsf {HP}}^{DR}_*(X) = H_{DR}^{\dim X - *}(X)$ is the de Rham cohomology of *X*. Indeed, by Examples 4.2 and 4.12, in this case $M(X,\mathfrak {g}) = \Omega _X$, the canonical right D-module of volume forms, and then ${\textsf {HP}}^{DR}_i(X) = H^{-i} \pi _* \Omega _X = H_{DR}^{\dim X - i}(X)$.

### Remark 5.3

The Poisson-de Rham homology is quite different, in general, from the ordinary Poisson homology. If *X* is an affine symplectic variety, it is true that ${\textsf {HP}}^{DR}_*(X) \cong {\textsf {HP}}_*(\mathcal {O}(X))$, both producing the de Rham cohomology of the variety. But when *X* is singular, if *X* has finitely many symplectic leaves, ${\textsf {HP}}^{DR}_*(X)$ can be nonzero only in degrees $-\dim X \le * \le \dim X$, since it is the pushforward of a holonomic $\mathcal {D}$-module on *X*. On the other hand, the ordinary Poisson homology ${\textsf {HP}}_*(\mathcal {O}(X))$ is in general nonzero in infinitely many degrees if *X* is singular and affine.

Theorem 4.7 (now valid for nonaffine *X*) together with Corollary A.19 implies:

### Corollary 5.4

If *X* is the union of finitely many $\mathfrak {g}$-leaves, then $H^{\mathfrak {g}-DR}_*(X)$ is finite dimensional. In particular, if *X* is a Poisson variety with finitely many symplectic leaves, then ${\textsf {HP}}^{DR}_*(X)$ is finite dimensional.

### Example 5.5

Suppose $\mathfrak {g}$ is any Lie algebra (or presheaf of Lie algebras) acting transitively on *X* (in particular, *X* is smooth), and assume *X* is connected. Then, as explained in Example 4.2, $M(X,\mathfrak {g})$, and hence $H^{\mathfrak {g}-DR}(X)$, is either zero or a line bundle (i.e., in the complex topology, there exist everywhere local sections invariant under (the flow of) $\mathfrak {g}$, which forms a line bundle because such sections are unique up to constant multiple). In the case that $M(X,\mathfrak {g})$ is a line bundle, the associated left D-module $L:=M(X,\mathfrak {g}) \otimes _{\mathcal {O}_X} \Omega _X^{-1}$ (for $\Omega _X$ the canonical bundle) has a canonical flat connection, and then $H^{\mathfrak {g}-DR}(X) = H_{DR}^{\dim X - i}(X,L)$, the de Rham cohomology with coefficients in *L*.

This vector bundle with flat connection need not be trivial. Consider [25, Example 2.38]: $X = (\mathbf C ^1{\setminus }\{0\}) \times \mathbf C ^1 = {\textsf {Spec}}\, \mathbf k [x,x^{-1},y]$ with $\mathfrak {g}$ the Lie algebra of vector fields preserving the multivalued volume form $d(x^{r}) \wedge dy$ for $r \in \mathbf k$. It is easy to check that this makes sense and that the resulting Lie algebra $\mathfrak {g}$ is transitive. Then, $M(X,\mathfrak {g})$ is the D-module whose local homomorphisms to $\Omega _X$ correspond to scalar multiples of this volume form, and hence $M(X,\mathfrak {g})$ is nontrivial (but with regular singularities) when *r* is not an integer. For $\mathbf k =\mathbf C$, the line bundle $L=M(X,\mathfrak {g})^\ell$ with flat connection has monodromy $e^{-2 \pi i r}$ going counterclockwise around the unit circle.

## Conjectures on symplectic resolutions

**6.1.** In the case where *X* is symplectic, by Example 5.2, ${\textsf {HP}}_0(\mathcal {O}(X)) \cong H^{\dim X}(X)$ and ${\textsf {HP}}^{DR}_i(X) \cong H^{\dim X - i}(X)$ for all *i*. It seems to happen that when ${\widetilde{X}} \rightarrow X$ is a symplectic resolution (see Example 2.12), we can also describe the Poisson-de Rham homology of *X* in the same way, and it coincides with that of ${\widetilde{X}}$. For notational simplicity, let us write $M(X) := M(X,\mathcal {O}_X)$ below. In this section, we set $\mathbf k = \mathbf C$.

### Conjecture 6.1

If $\rho : {\widetilde{X}} \rightarrow X$ is a symplectic resolution and *X* is affine, then:

- $${\textsf {HP}}_0(\mathcal {O}(X)) \cong H^{\dim X}({\widetilde{X}})$$;
- $${\textsf {HP}}_i^{DR}(X) \cong H^{\dim X - i}({\widetilde{X}})$$ for all *i*;
- $$M(X) \cong \rho _* \Omega _{{\widetilde{X}}}$$.

We remark first that (b) obviously implies (a), setting $i=0$. Next, (c) implies (b), since, for $\pi ^X: X \rightarrow \text {pt}$ and $\pi ^{{\widetilde{X}}}: \widetilde{X} \rightarrow \text {pt}$ the projections to points, we have $\pi ^X \circ \rho = \pi ^{{\widetilde{X}}}$. Thus, (c) implies that $\pi ^X_* M(X) = \pi ^X_* \rho _* \Omega _{{\widetilde{X}}} = \pi ^{{\widetilde{X}}}_* \Omega _{{\widetilde{X}}}$, whose cohomology is $H^{\dim {\widetilde{X}} - *}({\widetilde{X}}) = H^{\dim X - *}({\widetilde{X}})$.

Next, note that if one eliminates (a), the conjecture extends to the case where *X* is not necessarily affine. Indeed, part (c) is a local statement, so conjecture (c) for affine *X* implies the same conjecture for arbitrary *X* by taking an affine covering. As before, (c) implies (b) for arbitrary *X*.

Since (c) is a local statement, if it holds for $\rho : {\widetilde{X}} \rightarrow X$, then it follows that the same statement holds for slices to every symplectic leaf $Z \subseteq X$. Namely, recall that the Darboux–Weinstein theorem ([59]; see also [37, Proposition 3.3]) states that a formal neighborhood ${\hat{X}}_z$ of $z \in Z$, together with its Poisson structure, splits as a product ${\hat{Z}}_z \times X_Z$, for some formal transverse slice $X_Z$ to *Z* at *z*, which is unique (and independent of the choice of *z*) up to formal Poisson isomorphism. Now, for such a formal slice $X_Z$, letting $\rho ': {\widetilde{X}}_Z=\rho ^{-1}(X_Z) \rightarrow X_Z$ be the restriction of $\rho$, we obtain a formal symplectic resolution, and then the statement of (c) and hence also of (a) and (b) hold for $\rho '$. In the case that $X_Z$ is the formal neighborhood of the vertex of a cone *C* (expected to occur by [39, Conjecture 1.8]) and $\rho '$ is the restriction of a conical symplectic resolution $\rho _C: \widetilde{C} \rightarrow C$, this implies that Conjecture 6.1 holds for $\rho _C$ as well. Here and below a conical symplectic resolution is a $\mathbf C ^\times$-equivariant resolution for which the action on the base contracts to a fixed point (i.e., the base is a cone).

In particular, one can use this to compute ${\textsf {HP}}_0(\mathcal {O}(X_Z))$ and ${\textsf {HP}}_i^{DR}(X_Z)$ for all leaves *Z*. Conversely, [50, Theorem 4.1] shows that if one can establish the formal analogue of (a) for all $X_Z$, and if $\rho : {\widetilde{X}} \rightarrow X$ itself is a conical symplectic resolution, then Conjecture (c) follows for *X*.

### Remark 6.2

Since $\rho$ is semismall [37, Lemma 2.11], it follows from the decomposition theorem [2, Théorème 6.2.5] that $\rho _* \Omega _{{\widetilde{X}}}$ is a semisimple regular holonomic D-module on *X*. Moreover, by [50, Proposition 2.1], $\rho _* \Omega _{{\widetilde{X}}}$ is isomorphic to the semisimplification of a quotient $M(X)'$ of *M*(*X*). Conjecture 6.1 therefore states that $M(X) \cong M(X)'$ and that $M(X)'$ is semisimple. In the case that $\rho$ is a conical symplectic resolution, by [50, Proposition 3.6], $\rho _* \Omega _{{\widetilde{X}}}$ is actually rigid, which implies that any D-module whose semisimplification is $\rho _* \Omega _{{\widetilde{X}}}$ is already semisimple. Thus, in the conical case, $M(X)' \cong \rho _* \Omega _{{\widetilde{X}}}$, and Conjecture 6.1 states that in fact the quotient $M(X) \twoheadrightarrow M(X)'$ is an isomorphism. For more details on the quotient $M(X)'$, see Remark 6.8 below.

Conjecture 6.1 has been proved in many cases, with the notable exception of Nakajima quiver varieties.

### Remark 6.3

Let *X* be a Nakajima quiver variety. By [50, Theorem 4.1], since formal slices to all symplectic leaves of *X* are formal neighborhoods of Nakajima quiver varieties, to prove Conjecture 6.1 for *X*, it would suffice to prove part (a) for *X* and for all the quiver varieties that appear by taking slices. Thus, the full conjecture for the class of Nakajima quiver varieties would follow from part (a) for the class of Nakajima quiver varieties.

### Example 6.4

Let *Y* be a smooth symplectic surface. Then, one can set $X = {\textsf {Sym}}^n Y := Y^n / S_n$, the *n*-th symmetric power of *Y*. In this case one has the resolution $\rho : {\widetilde{X}} = {\text {Hilb}}^n Y \rightarrow X$. In this case, Conjecture 6.1(c) (and hence the entire conjecture) follows from [24, Theorem 1.17], which gives a direct computation of *M*(*X*) in this case; see Sect. 7 for more details.

### Example 6.5

Next suppose that $Y = \mathbf C ^2/\Gamma$, for $\Gamma < \textsf {SL}(2,\mathbf C )$ finite, is a du Val singularity, and $X := {\textsf {Sym}}^n Y$. Then, we can take the minimal resolution ${\widetilde{Y}} \rightarrow Y$. We obtain from the previous example the resolution $\rho _1: {\widetilde{X}} := {\text {Hilb}}^n {\widetilde{Y}} \rightarrow {\textsf {Sym}}^n {\widetilde{Y}}$, and we can compose this with $\rho _2: {\textsf {Sym}}^n {\widetilde{Y}} \rightarrow {\textsf {Sym}}^n Y$ to obtain the resolution $\rho = \rho _2 \circ \rho _1: {\widetilde{X}} \rightarrow X$. In this case, Conjecture 6.1 is proved in [24, Sect. 1.3], using the main result of [22] together with [32, Theorem 3]: see Sect. 7 for more details.

### Example 6.6

Suppose that *X* is the cone of nilpotent elements in a complex semisimple Lie algebra $\mathfrak {g}$. Let $\mathcal {B}$ be the flag variety, parameterizing Borel subalgebras of $\mathfrak {g}$. The cotangent fiber $T^*_{\mathfrak {b}} \mathcal {B}$ identifies with the annihilator of $\mathfrak {b}$ under the Killing form, i.e., the nilradical $[\mathfrak {b},\mathfrak {b}]$. Then, one has the Springer resolution $\rho : T^* \mathcal {B} \rightarrow X$, given by $\rho (\mathfrak {b},x) = x$. In this case, Conjecture 6.1 is a consequence of [34, Theorem 4.2 and Proposition 4.8.1(2)] (see also [47, Sect. 7]), as observed in [21].

### Example 6.7

Let *X* be as in the previous example. In this case the symplectic leaves are the nilpotent adjoint orbits $G \cdot e \subseteq X$, for *G* a semisimple complex Lie group with ${\textsf {Lie}}G = \mathfrak {g}$. Let $e \in \mathfrak {g}$ be a nilpotent element. One has a Kostant–Slodowy slice $S^0_e := X_{G \cdot e}$, transverse to $G \cdot e$, explicitly given by $S_e := e + \ker ({\text {ad}}f)$ and $S_e^0 := S_e \cap X$, where (*e*, *h*, *f*) is an $\mathfrak {sl}_2$-triple (whose existence is guaranteed by the Jacobson-Morozov theorem), equipped with a canonical Poisson structure, such that the formal neighborhood ${\hat{S}}_e$ of *e* is a formal slice. Let ${\widetilde{S}}_e^0$ be the preimage of $S^0_e$ under $\rho$. The Poisson algebra $\mathcal {O}(S_e^0)$ is called a *classical W-algebra*.

As observed above, the previous example implies that Conjecture 6.1 also holds for $\rho |_{{\widetilde{S}}_e^0}: {\widetilde{S}}_e^0 \rightarrow S_{e}^0$. In particular, one concludes that ${\textsf {HP}}_0(\mathcal {O}(S_e^0)) \cong H^{\dim S_e^0}({\widetilde{S}}_e^0)$ [21, Theorem 1.6], and the latter is the same as the top cohomology of the Springer fiber over *e*, $H^{\dim \rho ^{-1}(e)}(\rho ^{-1}(e))$, since $S_e$ and hence $S_e^0$ admits a contracting $\mathbf C ^\times$ action (the Kazhdan action) to $\rho ^{-1}(e)$ (explicitly, this is given on $S_e$ by $\lambda \cdot (x) = \lambda ^{2-h} x$).

More generally, we conclude also part (b) of Conjecture 6.1 for $S_e^0$, which yields in this case that ${\textsf {HP}}_i^{DR}(S_e^0) \cong H^{\dim \rho ^{-1}(e)-i}(\rho ^{-1}(e))$. By [21, Theorem 1.13], we can generalize even further and consider all of $S_e = e + \ker ({\text {ad}}f)$, and show that ${\textsf {HP}}_*^{DR}(S_e)$ is a (graded) vector bundle over $\mathfrak {g}//G \cong \mathfrak {h}/W$ with fibers given by the cohomology of the Springer fiber over *e*. Equivalently, we have the family of deformations $S_e^\eta := S_e \cap \chi ^{-1}(e)$ over $\mathfrak {g}//G$, with $\chi : \mathfrak {g}\rightarrow \mathfrak {g}//G$ the quotient; then, we conclude that $HP_i^{DR}(S_e^\eta )$ are all isomorphic to $H^{\dim \rho ^{-1}(e)-i}(\rho ^{-1}(e))$, for all $\eta \in \mathfrak {g}//G$ (the family ${\textsf {HP}}_i^{DR}(S_e^\eta )$ is flat over $\mathfrak {g}//G$).

### Remark 6.8

The deformation considered in Example 6.7 is part of a more general phenomenon. For a general projective symplectic resolution $\rho : {\widetilde{X}} \rightarrow X$, in [38], Kaledin proves that $\rho$ can be extended to a projective map $\rho :\widetilde{\mathcal {X}}\rightarrow \mathcal {X}$ of schemes over the formal disk $\Delta := {\textsf {Spec}}\,\mathbf C [[t]]$, such that, restricting to the point $0 \in \Delta$, we recover the original resolution $\rho : \widetilde{X} \rightarrow X$. Furthermore, he shows that $\mathcal {X}$ is normal and flat over $\Delta$, and that over the generic point, $\rho$ restricts to an isomorphism of smooth, affine, symplectic varieties [38, 2.2 and 2.5]. (In fact the construction provides more: for every choice of ample line bundle *L* on $\widetilde{X}$, there is a unique triple $(\widetilde{\mathcal {X}}, \mathcal {L},\omega _Z)$ up to isomorphism where $\mathcal {L}$ is a line bundle on $\widetilde{\mathcal {X}}$, $\omega _Z$ is a symplectic structure on the associated $\mathbf C ^\times$-torsor $Z \rightarrow \widetilde{\mathcal {X}}$, the $\mathbf C ^\times$ action is Hamiltonian for $\omega _Z$, the projection $Z \rightarrow \Delta$ is the moment map for the action, and the restrictions of $\mathcal {L}$ and $\omega _Z$ to ${\widetilde{X}}$ recover *L* and the original symplectic structure.) The family of maps over $\Delta$ (together with $\mathcal {L}$ and $\omega _Z$) is called a *twistor deformation*. In the case that $\rho$ is conical, we can moreover replace the formal disk $\Delta$ by the line $\mathbf C ={\textsf {Spec}}\mathbf C [t]$ and the map $\widetilde{\mathcal {X}} \rightarrow \mathcal {X}$ can be taken to be $\mathbf C ^\times$-equivariant.

In the general case (where $\rho$ need not be conical), let $X_t$ be the fiber of $\mathcal {X} \rightarrow \Delta$ over $t \in \Delta$ and $\widetilde{X_t}$ the fiber of $\widetilde{\mathcal {X}} \rightarrow \Delta$ over *t*. Then, one can show that Conjecture 6.1 implies that the family ${\textsf {HP}}_{DR}^i(X_t)$ is flat with fibers isomorphic to $H^{\dim X-i}({\widetilde{X}_t})$ (which is a vector bundle equipped with the Gauss–Manin connection). More generally, the conjecture for *X* implies that the family $M(X_t)$ of fiberwise D-modules is torsion-free: indeed, as explained in [50, Proposition 2.1], since $M(X_t) \cong \Omega _{X_t} \cong \rho _* \Omega _{{\widetilde{X}_t}}$ for generic *t*, the quotient $M(X)'$ of *M*(*X*) by the torsion of the family $M(X_t)$ is isomorphic to the semisimplification of $\rho _* \Omega _{{\widetilde{X}}}$. Conversely, if $\rho$ is conical, then as explained in Remark 6.2, we can replace the formal deformation by an actual $\mathbf C ^\times$-equivariant deformation over the line $\mathbf C$, and in this case [50, Proposition 3.6] implies that if the family $M(X_t)$ is torsion-free, then *M*(*X*) is already semisimple and the conjecture holds.

### Example 6.9

Next suppose that *X* is a conical Hamiltonian reduction of a symplectic vector space by a torus. Such a variety is called a hypertoric cone. More precisely, we can assume the symplectic vector space is a cotangent bundle, $V = T^* U$, and the torus is $G = (\mathbf C ^\times )^k$ for some $k \ge 1$, acting faithfully on *U* via $a: G \rightarrow \textsf {GL}(U)$, with the induced Hamiltonian action on *V* as in Example 2.15. Explicitly if $U=\mathbf C ^n$ for $n \ge k$, *a*(*G*) is a subgroup of the group of invertible diagonal matrices, and $(a_{ij})$ is the matrix of weights such that $a(\lambda _1,\ldots ,\lambda _k)(e_i) = \prod _{j=1}^k \lambda ^{a_{ij}} e_i$, then $\mu ((b_1,\ldots ,b_n),(c_1,\ldots ,c_n))= (\sum _{i=1}^n a_{ij} b_i c_i)_{j=1}^k$. Then, $X = \mu ^{-1}(0)//G$. In this case, for every character $\chi$ of *G*, we can form a GIT quotient ${\widetilde{X}} := \mu ^{-1}(0)//_\chi (G)$, mapping projectively to *X*. In the case this is a symplectic resolution, Conjecture 6.1 is proved in [50, Theorem 4.1, Example 4.6], by showing, as we mentioned, that Conjecture 6.1 follows (for conical symplectic resolutions) from its part (a) for slices to the symplectic leaves; since the slices in this case are also hypertoric cones, part (a) follows for these by [49].

### Remark 6.10

As noted in Remark 6.2, Conjecture 6.1 would imply that *M*(*X*) is regular and semisimple when *X* admits a symplectic resolution. This is not true for general *X*: we will explain in Remark 8.11 that already if *X* is a surface in $\mathbf C ^3$ which is a cone over a smooth curve in $\mathbf P ^2$, then *M*(*X*) is not semisimple unless the genus is zero (hence *X* is a quadric surface).

For regularity, [20, Example 4.11] gives a simple example where *M*(*X*) is not regular: let $X = Z \times \mathbf C ^2$ with *Z* is the surface $x_1^3+x_2^3+x_3^3=0$ in $\mathbf C ^3={\textsf {Spec}}\mathbf C [x_1,x_2,x_3]$. Using coordinates *p*, *q* on $\mathbf C ^2$, we consider the Poisson bracket given by $\{p,q\}=1$, $\{x_1,x_2\}=x_3^2$ (and cyclic permutations), and $\{q,f\}=0$, $\{p,f\}=|f|f$ for homogeneous $f \in \mathcal {O}(Z)$ of degree |*f*|. Then, *X* has two symplectic leaves: $X {\setminus } (\{0\} \times \mathbf C ^2)$ and $\{0\} \times \mathbf C ^2$. Now ${\textsf {HP}}_0(\mathcal {O}(Z))$ is a graded vector space (under $|x_1|=|x_2|=|x_3|=1$) of the form ${\textsf {HP}}_0(\mathcal {O}(Z)) \cong \mathbf C \oplus \mathbf C ^3[-1] \oplus \mathbf C ^3[-2] \oplus \mathbf C [-3]$ (a basis is given from monomials in $x_1,x_2,x_3$ of degree at most one in each variable). As a result, the algebraic flat connections on $\{0\} \times \mathbf C ^2$ given by $\nabla (f) = df - mf dp$, $m \in \{0,1,2,3\}$, all appear as quotients of *M*(*X*) (i.e., they admit sections which extend to Hamiltonian-invariant distributions on *X* supported on $\{0\} \times \mathbf C ^2$). As these connections have irregular singularities at $\{\infty \} = \mathbf P ^2 {\setminus } \mathbf C ^2$ for $m\ne 0$, we conclude that *M*(*X*) is not regular.

However, it is an open question whether, if *X* has finitely many symplectic leaves, *M*(*X*) must be *locally regular* on *X*, i.e., all composition factors are rational connections which have no irregular singularities in *X* itself. If this is true, then it would follow that *M*(*X*) is regular whenever *X* is proper (in particular, projective).

Let us remark that in the case when $\mathfrak {g} = {\textsf {Lie}}\, G$ and the action is the infinitesimal action associated with an action of *G* on *X* with finitely many orbits, then it is well known that *M*(*X*) is regular holonomic (see, e.g., [35, Section 5]). Note that, for general $\mathfrak {g}$, the action may not integrate to a group action, but formally locally it integrates to the action of a formal group; it would be interesting to try to use this and the argument of *op. cit.* to prove local regularity in general.

### Remark 6.11

There are many other interesting consequences of Conjecture 6.1 which would resolve open questions. For instance, the conjecture implies that every symplectic resolution of *X* is strictly semismall in the following sense: for every symplectic resolution $\rho : {\widetilde{X}} \rightarrow X$ and every symplectic leaf $Y \subseteq X$, one has $\dim \rho ^{-1}(Y) = \frac{1}{2}(\dim X + \dim Y)$. The semismallness condition itself is equivalent to the inequality $\le$. This corollary follows because, whenever *X* has finitely many symplectic leaves, the intersection cohomology D-module of every symplectic leaf closure (i.e., intermediate extension of the canonical right D-module on the leaf itself) is a composition factor of *M*(*X*) (which follows from [20, Sect. 4.3]; see also [25, Propositions 2.14 and 2.24]), and there is a composition factor of $\rho _* \Omega _{{\widetilde{X}}}$ with support equal to the leaf closure if and only if the dimension equality holds. Another interesting potential application (pointed out to us by D. Kaledin) is a conjecture variously attributed to Demailly, Campana, and Peternell [39, Conjecture 1.3] that if $T^*Z \rightarrow Y$ is a symplectic resolution of an affine variety *Y*, then *Z* is a partial flag variety. Namely the conjecture implies that the maximal ideal $\mathfrak {m}_0 \subseteq \mathcal {O}(Y)$ of the origin is a perfect Lie algebra; the conjecture would follow if one shows that $Z = G/P$ where ${\textsf {Lie}}G \subseteq \mathfrak {m}_0$ is the degree-one subspace and *P* is a parabolic subgroup of *G*.

## Symmetric powers and Hilbert schemes

In this section we would like to discuss results from [24] on the zeroth Poisson homology of symmetric powers. We continue to set $\mathbf k =\mathbf C$. In this section, the variety *Y* will always be assumed to be connected.

### The main results

Given an affine variety $Y = {\textsf {Spec}}A$, let $S^n Y := Y^n / S_n = {\textsf {Spec}}\, {\textsf {Sym}}^n A$ be the *n*-th symmetric power of *Y*. Let the symbol & denote the product in the symmetric algebra. Note that $\bigoplus _{n \ge 0} {\textsf {HP}}_0(\mathcal {O}(S^n Y))^*$ is a graded algebra, with multiplication induced, via the inclusions ${\textsf {HP}}_0(\mathcal {O}(X))^* \subseteq \mathcal {O}(X)^*$, by the maps $\mathcal {O}(S^m Y)^* \otimes \mathcal {O}(S^n Y)^* \rightarrow \mathcal {O}(S^{m+n} Y)^*$ dual to the symmetrization maps $\mathcal {O}(S^{m+n}Y) \rightarrow \mathcal {O}(S^m Y) \otimes \mathcal {O}(S^n Y)$ sending *f* to the function

$$\begin{aligned} ((x_1,\ldots ,x_m),(x_{m+1},\ldots ,x_{m+n})) \mapsto \frac{1}{(m+n)!} \sum _{\sigma \in S_{m+n}} f(x_{\sigma (1)},\ldots ,x_{\sigma (m+n)}). \end{aligned}$$

To see that this indeed induces maps on Poisson traces (${\textsf {HP}}_0^*$), note that $\mathcal {O}(Y)$ acts on $\mathcal {O}(S^n Y) = {\textsf {Sym}}^n \mathcal {O}(Y)$ by Lie bracket, and ${\textsf {HP}}_0(\mathcal {O}(S^n Y))^* = (\mathcal {O}(S^n Y)^*)^{\mathcal {O}(S^n Y)} = (\mathcal {O}(S^n Y)^*)^{\mathcal {O}(Y)}$. Then, it remains to observe that the maps above are compatible with this adjoint action of $\mathcal {O}(Y)$, so they indeed induce bilinear maps as claimed on Poisson traces, which are easily seen to be associative with unit $1 \in {\textsf {HP}}_0(\mathcal {O}(S^0Y))={\textsf {HP}}_0(\mathbf C )=\mathbf C$.

#### Theorem 7.1

[24, Theorem 1.1] Let *Y* be an affine symplectic variety. Then, there is a canonical isomorphism of graded algebras,

7.1 $$ \begin{aligned}&{\textsf {Sym}}({\textsf {HP}}_0(\mathcal {O}(Y))^*[t]) \mathop {\rightarrow }^\sim \bigoplus _{n \ge 0} {\textsf {HP}}_0(\mathcal {O}(S^n Y))^*, \nonumber \\&\phi \cdot t^{m-1} \mapsto \Bigl ( (f_1 \& \cdots \& f_m) \mapsto \phi (f_1 \cdots f_m) \Bigr ), \end{aligned}$$

where the grading is given by $|{\textsf {HP}}_0(\mathcal {O}(S^n Y))^*| = n$ (on both sides of the isomorphism), and $|t| = 1$.

If we expand the symmetric algebra on the LHS in (7.1) and dualize, we explicitly obtain the following. Recall that a partition of *n* of length *k* is a tuple $\lambda = (\lambda _1, \ldots , \lambda _k)$ such that $\lambda _1 \ge \lambda _2 \ge \cdots \ge \lambda _k \ge 1$ and $\lambda _1 + \cdots + \lambda _k = n$. If $\lambda$ is a partition of *n*, we write $\lambda \vdash n$, and let $|\lambda |$ denote its length. Let $S_\lambda < S_{|\lambda |}$ be the subgroup preserving the partition $\lambda$. Explicitly, $S_{\lambda } = S_{r_1} \times \cdots \times S_{r_k}$ where, for all *j*,

$$\begin{aligned} \lambda _{r_1+\cdots +r_j} > \lambda _{r_1+\cdots +r_j+1} = \lambda _{r_1 + \cdots + r_j + 2} = \cdots = \lambda _{r_1 + \cdots + r_j + r_{j+1}}. \end{aligned}$$

Then, (7.1) states that, for all $n \ge 1$,

7.2 $$\begin{aligned} {\textsf {HP}}_0(\mathcal {O}(S^n Y)) \cong \bigoplus _{\lambda \vdash n} ({\textsf {HP}}_0(\mathcal {O}(Y))^{\otimes |\lambda |})_{S_\lambda }. \end{aligned}$$

Next, it is well known that if *Y* is connected, then ${\textsf {HP}}_0(\mathcal {O}(Y)) \cong H^{\dim Y}(Y)$, the top cohomology of *Y*, via the isomorphism $[f] \mapsto f \cdot {\text {vol}}_Y$, where ${\text {vol}}_Y$ is the canonical volume form (i.e., the $\frac{1}{2} \dim Y$-th exterior power of the symplectic form). We can write the above more explicitly using the coefficients $a_n(i)$ which give the number of *i*-multipartitions of *n* (i.e., collections of *i* ordered partitions whose sum of sizes is *n*), i.e.,

7.3 $$\begin{aligned} \prod _{m \ge 1} \frac{1}{(1-t^m)^i} = \sum _{n \ge 0} a_n(i) \cdot t^n. \end{aligned}$$

#### Corollary 7.2

[24, Corollary 1.2] If *Y* is a symplectic variety, then $\dim {\textsf {HP}}_0(\mathcal {O}(S^n Y)) = a_n(\dim H^{\dim Y}(Y))$.

#### Notation 7.3

Whenever we take tensor products (so also symmetric powers) of $\mathbf C \llbracket \hbar \rrbracket$-algebras complete in the $\hbar$-adic topology (e.g., ${\textsf {Sym}}^n A_\hbar$), we mean the $\hbar$-adic completion of the usual tensor product (and hence symmetric power).

#### Notation 7.4

When *B* is a $\mathbf C \llbracket \hbar \rrbracket$-algebra complete in the $\hbar$-adic topology, let $\textsf {HH}_0(B) := B / \overline{[B,B]}$ (i.e., we take the closure, equivalently $\hbar$-adic completion, of [*B*, *B*]).

The results above imply the degeneration of the spectral sequence computing the zeroth Hochschild homology of quantizations of $S^nY$. Let *Y* be an affine symplectic variety, and let $A_\hbar$ be any deformation quantization of $\mathcal {O}(Y)$, so that ${\textsf {Sym}}^n A_\hbar$ is a deformation quantization of $\mathcal {O}(S^n Y)$). Then, the spectral sequence associated with the deformation yields a natural $\mathbb {C}((\hbar ))$-linear surjection

$$\begin{aligned} \theta : {\textsf {HP}}_0(\mathcal {O}(S^n Y))((\hbar )) \twoheadrightarrow {\textsf {gr}}\textsf {HH}_0 \left( {\textsf {Sym}}^n A_\hbar [\hbar ^{-1}]\right) , \end{aligned}$$

where the filtration on $\textsf {HH}_0({\textsf {Sym}}^n A_\hbar [\hbar ^{-1}])$ is induced by the filtration of ${\textsf {Sym}}^n A_\hbar [\hbar ^{-1}]$ by powers of $\hbar$, and ${\textsf {gr}}$ denotes the $\hbar$-adically completed associated graded space.

#### Corollary 7.5

[24, Corollary 1.3] $\theta$ is an isomorphism.

Namely, Corollary 7.5 follows from Corollary 7.2 and the computation of $\textsf {HH}_0({\textsf {Sym}}^nA_\hbar [\hbar ^{-1}])$ from [19], which jointly show that $\dim {\textsf {HP}}_0({\mathcal {O}}(S^nY))=\dim \textsf {HH}_0({\textsf {Sym}}^nA_\hbar [\hbar ^{-1}])$.

### Sketch of proof of Theorem 7.1

The proof of Theorem 7.1 is based on the following theorem, giving the structure of *M*(*X*) when $X = S^n Y$, for *Y* a symplectic variety that need not be affine. Let $\Delta _i: Y \hookrightarrow S^i Y$ be the diagonal embedding, and for $\sum _{j=1}^k r_j i_j = n$, let $q: (S^{i_1} Y)^{r_1} \times \cdots \times (S^{i_k} Y)^{r_k} \twoheadrightarrow S^n Y$ be the obvious projection.

#### Theorem 7.6

[24, Theorem 1.17]

7.4 $$\begin{aligned} M(S^n Y)\cong & {} \bigoplus _{r_1 \cdot i_1 + \cdots + r_k \cdot i_k = n, 1 \le i_1< \cdots < i_k, r_j \ge 1\, \forall j} \nonumber \\&q_* \bigl ((\Delta _{i_1})_*(\Omega _Y)^{\boxtimes r_1} \boxtimes \cdots \boxtimes (\Delta _{i_k})_*(\Omega _Y)^{\boxtimes r_k} \bigr )^{S_{r_1} \times \cdots \times S_{r_k}}. \end{aligned}$$

Indeed, Theorem 7.1 (at the level of vector spaces) is obtained from Theorem 7.6 by computing the direct image of *M*(*X*) to the point, and it is not hard to check that the corresponding isomorphism of vector spaces is an algebra map.

### Sketch of proof of Theorem 7.6

Now let us say a few words about the proof of Theorem 7.6. One can show that all the simple summands *S*, on the right-hand side, are composition factors of *M*(*X*) (by constructing surjections $M(X)|_U\rightarrow S|_U$ on dense open sets *U* in *X*), so it suffices to show that: (a) they are the only composition factors, which furthermore occur with multiplicity 1, and (b) *M*(*X*) is semisimple.

We first show that (a) implies (b). To do this we need to work on $Y^n$ rather than on $X=S^n Y = Y^n/S_n$. Let $p: Y^n \rightarrow S^n Y=X$ be the projection. By definition, $M(X) \cong p_* ({\widetilde{M}})^{S_n}$ where ${\widetilde{M}} := M(Y^n,\mathcal {O}(Y^n)^{S_n})$ is a right D-module on $Y^n$, which is also holonomic since the $\mathcal {O}(Y^n)^{S_n}$-leaves of $Y^n$ are the diagonals (subvarieties $Z \subseteq Y^n$ obtained by setting certain components to be equal). Similarly, the summands *S* in Theorem 7.6 are of the form $S \cong p_*({\widetilde{S}})^{S_n}$ for some D-modules ${\widetilde{S}}$ on $Y^n$. In fact, each ${\widetilde{S}}$ is the pushforward of the canonical right D-module $\Omega _{Z}$ for some diagonal $Z \cong Y^m \subseteq Y^n, m \le n$ under the embedding $Z \rightarrow Y^n$. Since the ${\widetilde{S}}$ are also composition factors of ${\widetilde{M}}$, to deduce (b) from (a), it suffices to show that ${\text {Ext}}^1({\widetilde{S}}, \widetilde{S'})=0$ for distinct $S,S'$. The characteristic variety of each ${\widetilde{S}}$ is the conormal bundle $T^*_Z Y$ of the associated diagonal $Z \subseteq Y^n$, and for distinct diagonals the intersection of these conormal bundles has codimension at least $\dim Y \ge 2$ (i.e., the dimension is at most $(n-1)\dim Y$). By a well-known result from D-module theory ([44, Theorem 1.2.2], see also [45, 1.4]), this implies that ${\text {Ext}}^1({\widetilde{S}}, \widetilde{S'}) = 0$. (For a slightly different argument avoiding [44, Theorem 1.2.2], see [24], Lemma 2.1).

To prove (a), it suffices to replace $S^nY$ with the formal neighborhood of a point of the diagonal in $S^nY$. In other words, by the formal Darboux theorem, it is sufficient to consider the flat case, when $Y={\widehat{V}}$ is the formal neighborhood of zero in a symplectic vector space *V*. In this case, by Example 4.8, we only have to show that each multiplicity space for the intersection cohomology D-module of each diagonal is one-dimensional for all $m \le n$. By induction on *n* we can restrict to the delta-function D-module of the origin. Then, since $M(S^n V)$ is semisimple, it suffices to show that

7.5 $$\begin{aligned} {\text {Hom}}_{{\mathcal {D}}(S^n Y)}(M(S^n Y), \delta _{Y}) \cong \mathbf C . \end{aligned}$$

Finally, (7.5) can be restated without using ${\mathcal {D}}$-modules in the form of the following lemma, which plays a central role in the proof, and concludes our sketch of it:

#### Lemma 7.7

[24, Lemma 2.3] The space of symmetric polydifferential operators $\psi : \mathcal {O}(V)^{\otimes (n-1)} \rightarrow \mathcal {O}(V)$ invariant under Hamiltonian flow is one dimensional, and spanned by the multiplication map. The same holds for polydifferential operators on the completion $\widehat{\mathcal {O}(V)}=\mathcal {O}({\widehat{V}})$ of $\mathcal {O}(V)$ with respect to the augmentation ideal.

#### Proof

It suffices to pass to the formal completion and consider polydifferential operators on $\widehat{\mathcal {O}(V)}$. Such polydifferential operators are determined by their value on elements $f^{\otimes (n-1)}$ for $f \in \widehat{\mathcal {O}(V)}$, since they are symmetric and hence determined by their restriction to ${\textsf {Sym}}^{n-1} \widehat{\mathcal {O}(V)}$. Furthermore, we can assume that $f'(0) \ne 0$, since the complement of this locus in the pro-vector space $\widehat{\mathcal {O}(V)}$ has codimension equal to $\dim V \ge 2$.

Write $V \cong \mathbf C ^{2n}$ with the standard symplectic form $\omega = \sum _{i=1}^{\dim V} \mathrm{d}x_i \wedge dy_i$. Applying the formal Darboux theorem, there is a formal symplectomorphism of *V* whose pullback takes *f* to $x_1$ (i.e., *f* can be completed to a coordinate system in which the symplectic form is the standard one), so we can assume $f=x_1$. Since all formal symplectic automorphisms are obtained by integrating Hamiltonian vector fields, it suffices to consider the value $\psi (x_1^{\otimes (n-1)})$. This value must be a function that, in coordinates, depends only on $x_1$, since such functions are the only ones which are invariant under all symplectic automorphisms fixing $x_1$. By linearity and invariance under conjugation by rescaling $x_1$ (and applying the inverse scaling to $y_1$), we deduce that $\psi (x_1^{\otimes (n-1)}) = \lambda \cdot x_1^{n-1}$ for some $\lambda \in \mathbf C$. Thus, on $x_1^{\otimes (n-1)}$, $\psi$ coincides with $\lambda$ times the multiplication operator, $f_1 \otimes \cdots \otimes f_{n-1} \mapsto \lambda f_1 \cdots f_{n-1}$. The latter operator is evidently symmetric and invariant under Hamiltonian flow. On the other hand, we have argued that a symmetric operator invariant under Hamiltonian flow is uniquely determined by its value on $x_1^{\otimes (n-1)}$. So $\psi$ is equal to $\lambda$ times the multiplication operator, as desired. $\square$

As a by-product, we obtain the following theorem:

#### Theorem 7.8

[24, Theorem 1.6] Let *V* be a finite-dimensional symplectic vector space, and realize $V^{n-1}$ as the set of elements $(v_1 \ldots ,v_n)\in V^n$ such that $\sum _{i=1}^{n}v_i=0$. Then, ${\textsf {HP}}_0(\mathcal {O}(V^{n-1} / S_n))^* \cong \mathbf C$ spanned by the augmentation map $\mathcal {O}(V^{n-1}) \rightarrow \mathbf C$. In other words, the Lie algebra $\mathfrak {g}$ of $S_n$-invariant Hamiltonian vector fields on $V^{n-1}$ is perfect: $\mathfrak {g}=[\mathfrak {g},\mathfrak {g}]$.

## Structure of *M*(*X*) for complete intersections with isolated singularities

In this section we discuss results from [25, Sect. 5] and [23] concerning *M*(*X*) and ${\textsf {HP}}^{DR}_*(X)$ when *X* is a complete intersection surface with isolated singularities (or more generally of arbitrary dimension, if one suitably defines the Lie algebra $\mathfrak {g}$ of Hamiltonian vector fields). In particular, we will recover topological information about the singularities, including the Milnor numbers and genera, and find examples where *M*(*X*) is not semisimple. For concreteness, we will take *X* to be a surface in $\mathbf C ^3$ throughout most of the section and explain at the end how the arguments extend to general (locally) complete intersections (of arbitrary dimension) in complex affine space.

### Main results for surfaces in $\mathbf C ^3$

Let $X = \{f=0\} \subseteq \mathbf C ^3={\textsf {Spec}}\, \mathbf C [x_1,x_2,x_3]$ be a surface with *f* irreducible. It is naturally Poisson, with bracket $\{x_1,x_2\} = f_{x_3}$ together with cyclic permutations of the indices. We will be interested particularly in *f* having two nice properties:

#### Definition 8.1

A variety *X* is said to have isolated singularities if the singular locus of *X* is finite. A function $f \in \mathbf C [x_1,\ldots ,x_n]$ is said to have isolated singularities if $\{f=0\}$ is a variety with isolated singularities.

#### Definition 8.2

A function $f \in \mathbf C [x_1,\ldots ,x_n]$ is quasi-homogeneous of weight $|f|=m \ge 1$ with respect to weights $|x_i|=a_i \ge 1$ if *f* is a linear combination of monomials of degree *m* with respect to these weights.

Note that *f* is quasi-homogeneous if and only if $X=\{f=0\}$ is conical with respect to the $\mathbf C ^\times$ action $\lambda \cdot (x_1,\ldots ,x_n) = (\lambda ^{a_1} x_1, \ldots , \lambda ^{a_n} x_n)$, i.e., this action (which contracts to the origin) preserves *X*. Note that if *f* is quasi-homogeneous with isolated singularities, then since the singular locus is preserved under the action of $\mathbf C ^\times$, it must be identically $\{0\}$ (or empty).

#### Example 8.3

In the case that $f \in \mathbf C [x_1,x_2,x_3]$ is quasi-homogeneous with weight *m* with respect to $|x_i|=a_i$, we see that the Poisson bracket has degree $d:=m-(a_1+a_2+a_3)$. If moreover *f* has isolated singularities (i.e., its singular locus is $\{0\}$), then *X* is well known to be isomorphic to a du Val singularity, $\mathbf C ^2/\Gamma$ for $\Gamma < \textsf {SL}(2,\mathbf C )$ a finite subgroup, if $d < 0$; to have a simple elliptic singularity at the origin if $d=0$; and to have neither a du Val nor elliptic singularity if $d > 0$. In particular, up to isomorphism, in the case $d < 0$ (a du Val singularity) *X* is isomorphic to one of the following (see, e.g., [10] and [17, Proposition 2.3.2]):

8.1 $$\begin{aligned} A_{m-1}{:}~\Gamma= & {} \mathbf Z /m,\quad a_1=2,\quad a_2=a_3=m,\quad f = x_1^m + x_2^2 + x_3^2, \end{aligned}$$

8.2 $$\begin{aligned} D_{m+2}{:}~ \Gamma= & {} \widetilde{D_{2m}},\quad a_1=2,\quad a_2=m,\quad a_3=m+1, f = x_1^{m+1} + x_1 x_2^2 + x_3^2, \end{aligned}$$

8.3 $$\begin{aligned} E_6{:}~ \Gamma= & {} \widetilde{A_4},\quad a_1=3,\quad a_2=4,\quad a_3=6,\quad f = x_1^4 + x_2^3 + x_3^2, \end{aligned}$$

8.4 $$\begin{aligned} E_7{:}~ \Gamma= & {} \widetilde{S_4},\quad a_1=4,\quad a_2=6,\quad a_3=9,\quad f = x_1^3 x_2 + x_2^3 + x_3^2, \end{aligned}$$

8.5 $$\begin{aligned} E_8{:}~ \Gamma= & {} \widetilde{A_5},\quad a_1=6,\quad a_2=10,\quad a_3=15,\quad f = x_1^5 + x_2^3 + x_3^2; \end{aligned}$$

and in the case $d=0$ (elliptic), then *X* is isomorphic to one of the following forms:

8.6 $$\begin{aligned} {\widetilde{E}_6}{:}~ a_1= & {} a_2=a_3=1,\quad f= x_1^3 + x_2^3 + x_3^3 + \lambda x_1x_2x_3, \end{aligned}$$

8.7 $$\begin{aligned} {\widetilde{E}_7}{:}~ a_1= & {} a_2=1,\quad a_3=2,\quad f = x_1^4 + x_2^4 + x_3^2 + \lambda x_1x_2x_3, \end{aligned}$$

8.8 $$\begin{aligned} {\widetilde{E}_8}{:}~ a_1= & {} 1,\quad a_2=2,\quad a_3=3,\quad f = x_1^6 + x_2^3 + x_3^2 + \lambda x_1x_2x_3. \end{aligned}$$

#### Notation 8.4

For a possibly singular complex algebraic or analytic variety *X*, let $H^*_{{\textsf {top}}}(X)$ denote the topological cohomology of *X*.

#### Notation 8.5

For $s \in X$ an isolated singularity, let $\mu _s$ denote the Milnor number at *s*. Let $g_s$ denote the “reduced genus” of the singularity at *s*, which we define as $g_s = \dim H^{\dim X-1}(Y, \mathcal {O}_Y)$, for $\rho : {\widetilde{X}} \rightarrow X$ any resolution of singularities and $Y = \rho ^{-1}(s)$ (this definition does not depend on the choice of resolution).

By [33, 48], if $X_t$ is a smoothing of *X*, then for *B*(*s*) a small ball about *s*, $X_t \cap B(s)$ is homotopic to a bouquet of $\mu _s$ spheres of dimension $\dim X$. In the case *X* is a hypersurface in $\mathbf C ^n$, the Milnor number can be described as the codimension in $\mathcal {O}(X)$ of the ideal generated by the partial derivatives of *f*.

#### Theorem 8.6

[23, Theorem 2.4] If *X* is a surface in $\mathbf C ^3$ with isolated singularities $s_1,\ldots ,s_k$, then ${\textsf {HP}}^{DR}_*(X) \cong H_{{\textsf {top}}}^{2-*}(X) \oplus \bigoplus _{i=1}^k\mathbf C ^{\mu _{s_i}}$, placing $\mathbf C ^{\mu _{s_i}}$ in degree zero.

Theorem 8.6 follows from the following structure theorem for *M*(*X*) in this case. Let $Z \subseteq X$ be the singular locus and $j: X{\setminus } Z \rightarrow X$ the open embedding. Recall that, for *M* a holonomic right D-module on $X{\setminus } Z$, there are pushforward complexes of D-modules $j_! M, j_* M$ on *X* and a holonomic D-module $j_{!*} M$ on *X* satisfying the adjunction properties ${\text {Hom}}(H^0j_! M, N) = {\text {Hom}}(M, j^! N)$ and ${\text {Hom}}(N,H^0j_* M) = {\text {Hom}}(j^! N,M)$ (for $j^! N = j^* N$ the restriction of *N* to the open subset *U*); also $j_{!*} M$ is the minimal extension, which means for example that $j_{!*} M$ is simple if and only if *M* is. Since $X {\setminus } Z$ is a smooth symplectic variety, $j^! M(X) = \Omega _{X {\setminus } Z}$, which implies that *M*(*X*) has a composition series one of whose composition factors is the intersection cohomology D-module of *X*, ${\text {IC}}(X) = j_{!*} \Omega _{X {\setminus Z}}$, and the others which are D-modules supported on the singular locus, and hence are delta-function D-modules since the singular locus is finite. Let $M(X)_{\text {ind}}$ be the indecomposable summand of *M*(*X*) which has ${\text {IC}}(X)$ as a composition factor.

#### Theorem 8.7

[23, Theorem 2.7] Let $X = \{f=0\} \subseteq \mathbf C ^3$ have singular locus $Z = \{s_1, \ldots , s_k\}$ (with the $s_i$ distinct). Then there is a short exact sequence

$$\begin{aligned} 0 \rightarrow H^0j_! \Omega _{X{\setminus } Z} \rightarrow M(X) \rightarrow \bigoplus _{i=1}^k \delta ^{\mu _{s_i}} \rightarrow 0. \end{aligned}$$

#### Conjecture 8.8

[23, Conjecture 3.8]^1^ Under the same hypotheses as in Theorem 8.7, we have a short exact sequence

$$\begin{aligned} 0 \rightarrow H^0j_! \Omega _{X {\setminus } Z} \rightarrow M(X)_{\text {ind}} \rightarrow \bigoplus _i \delta ^{g_{s_i}} \rightarrow 0. \end{aligned}$$

#### Theorem 8.9

[23, Proposition 3.11] Conjecture 8.8 is true if *f* is quasi-homogeneous.

#### Remark 8.10

Theorem 8.7 shows that there are delta-function submodules of *M*(*X*) which are not direct summands whenever $H^0 j_! \Omega _{X{\setminus } Z} \ncong {\text {IC}}(X)$, the minimal extension of $\Omega _{X{\setminus } Z}$. Equivalently, this holds whenever ${\text {Ext}}^1({\text {IC}}(X),\delta _s) \ne 0$ for some point $s \in Z$. Since ${\text {Ext}}^1({\text {IC}}(X),\delta _s) \cong {\text {Ext}}^1(\delta _s, {\text {IC}}(X))^*$ by Verdier duality, we actually see that *M*(*X*) is not semisimple whenever possible, i.e., whenever there exists a nonsemisimple extension of $\Omega _{X{\setminus } Z}$ to *X*. One can compute that ${\text {Ext}}^1({\text {IC}}(X),\delta _s) \cong H_{{\textsf {top}}}^1(U_s {\setminus } \{s\})$ where $U_s$ is some contractible neighborhood of *s* (see [14, Lemma 3.5], similar to [20, Lemma 4.3]), which exists by [27], cf. also [48, 2.10]. Here $U_s {\setminus }\{s\}$ is homotopic to the topological *link* of *s*. Thus, we see that *M*(*X*) is not semisimple whenever the link has nonzero first Betti number.

#### Remark 8.11

Taking the special case where *X* is a cone over a smooth curve $\Sigma$ in $\mathbf P ^2$ of degree *d*, recall that the reduced genus of the singularity is $g = \frac{(d-1)(d-2)}{2}$ (the same as the genus of $\Sigma$) and the Milnor number is $\mu =(d-1)^3$. In this case, we see that $H_{{\textsf {top}}}^1(X {\setminus } \{0\}) \cong H_{{\textsf {top}}}^1(\Sigma )$ (since $X{\setminus } \{0\}$ is homotopic to a nontrivial $S^1$-bundle over $\Sigma$). Therefore, by Remark 8.10 we obtain that $H^0 j_! \Omega _{X {\setminus } \{0\}}$ is an extension given by a short exact sequence of the form

8.9 $$\begin{aligned} 0 \rightarrow \delta _0^{2g} \rightarrow H^0 j_! \Omega _{X {\setminus } \{0\}} \rightarrow {\text {IC}}(X) \rightarrow 0. \end{aligned}$$

We obtain from Theorem 8.9 that $M(X)_{\text {ind}}$ has a filtration with subquotients $\delta ^{2g}, {\text {IC}}(X)$, and $\delta ^g$, in that order. In particular, for $g > 0$, it is not semisimple, and moreover not self-dual (there is twice the multiplicity of $\delta$ function D-modules on the bottom as on the top). From Theorem 8.7 we see that $M(X) \cong M(X)_{\text {ind}} \oplus \delta _0^{\mu -g}$.

### The family $M(X_t)$ of fiberwise D-modules on a smoothing

The results can be reinterpreted in terms of a smoothing. Namely, we can consider all the level sets $X_t := \{f=t\} \subseteq \mathbf C ^3$ of *f* in $\mathbf C ^3$ as $t \in \mathbf C$ varies, which for generic *t* will be smooth. It is then well known that, for generic *t*, $\dim H_{{\textsf {top}}}^2(X_t) = \dim H_{{\textsf {top}}}^2(X) + \sum _{s \in Z} \mu _s$, where again $Z \subseteq X$ is the singular locus which we assume is finite; this is a consequence of the fact from [33, 48] that, for every $s \in Z$ and $0 < |t| \ll 1$, the intersection of $X_t$ with a small ball about $s \in \mathbf C ^3$ is homotopic to a bouquet of $\mu _s$ 2-spheres. As a result, Theorem 8.6 implies that $\dim {\textsf {HP}}_0(\mathcal {O}(X)) = \dim H_{{\textsf {top}}}^2(X_t)$ for generic *t*. Since $X_t$ is a smooth symplectic surface, $H_{{\textsf {top}}}^2(X_t) \cong {\textsf {HP}}_0(\mathcal {O}(X_t))$ (by Example 5.2). Thus, near $t=0$ the family ${\textsf {HP}}_0(X_t)$ of vector spaces has constant dimension, i.e., forms a vector bundle. We have proved:

#### Corollary 8.12

[23, Corollary 1.4] Assume $X = \{f=0\} \subseteq \mathbf C ^3$ has isolated singularities. Then, the sheaf ${\textsf {HP}}_0(\mathcal {O}(X_t))$ on $\mathbf C$ is a vector bundle near $t=0$ of rank $\dim H_{{\textsf {top}}}^2(X) + \sum _{s \in Z} \mu _s = \dim {\textsf {HP}}_0(\mathcal {O}(X))$. The generic fiber is $H_{{\textsf {top}}}^2(X_t)$.

The entire Poisson-de Rham homology similarly forms a graded vector bundle [23, Corollary 2.5], since the dimension of $H_{{\textsf {top}}}^i(X_t)$ does not depend on *t* for $i \in \{0,1\}$, and this equals the dimension of ${\textsf {HP}}^{DR}_{2-i}(X)$.

In fact, this is a shadow of a similar phenomenon for the D-modules $M(X_t)$, which is central to the proof of the main results. Note that, for each $t \in \mathbf C$, we can consider the D-module $M(X_t)$ on $X_t$, which for $i_t: X_t \rightarrow \mathbf C ^3$ the embedding, is realized as $(i_t)_\natural M(X_t)$, a right $\mathcal {D}(\mathbf C ^3)$-module on $\mathbf C ^3$ supported on $X_t$.

#### Theorem 8.13

[23, Theorem 2.8] The family on $\mathbf C$ of right $\mathcal {D}(\mathbf C ^3)$-modules with fiber $(i_t)_\natural M(X_t)$ at $t \in \mathbf C$ is flat near $t=0$. For generic *t*, the fiber of this family is the right D-module is isomorphic to $(i_t)_\natural \Omega _{X_t}$.

Applying the pushforward functor $\pi _*$, for $\pi : \mathbf C ^3 \rightarrow \text {pt}$ the projection to a point, Theorem 8.13 recovers Corollary 8.12.

### Sketch of proof of Theorems 8.6, 8.7 and 8.13

Theorem 8.6 follows immediately from Theorem 8.7. For the latter, since *M*(*X*) is locally defined, we can restrict to a neighborhood $U_s$ of each singular point $s \in Z$, and if we work in the analytic category, we can take the neighborhood to be contractible ([27], cf. also [48, 2.10]). Moreover, in this case we can show [25, Corollary 5.9] (using results of G.-M. Greuel [31]) that the maximal quotient of $M(U_s)$ supported at the singularity is $\delta ^{\mu _s}$, or equivalently [25, Theorem 5.11] that ${\textsf {HP}}_0(\mathcal {O}(U_s)) \cong \mathbf C ^{\mu _s}$. The main idea is to compare ${\textsf {HP}}_0(\mathcal {O}(U_s))$ directly with the cohomology of the de Rham complex of $U_s$ modulo those differential forms which are torsion (i.e., restrict to zero on $U_s {\setminus } \{s\}$). This takes care of the last term of the sequence in Theorem 8.7.

Theorem 8.7 then reduces to the statement that the canonical map $H^0j_! \Omega _{X{\setminus } Z} \rightarrow M(X)$ (obtained by adjunction from $\Omega _{X {\setminus } Z} \cong j^!M(X) = M(X)|_{X {\setminus } Z}$) is injective. We then prove this and Theorem 8.13 simultaneously. The argument is as follows: in general, if $M(X_t)$ is not torsion-free, then for each *t* we can consider the torsion at *t*, $M(X_t)_{{\mathrm {tor}}}$, such that $M(X_t)' := M(X_t)/M(X_t)_{{\mathrm {tor}}}$ is torsion-free. Note that $M(X_t)_{{\mathrm {tor}}}$ is zero for generic *t*. Applying the pushforward functor $\pi _*$ for $\pi : \mathbf C ^3 \rightarrow \text {pt}$ the projection to a point, we can see that $\pi _* M(X_t)'$ is quasi-isomorphic to a complex of finitely-generated and torsion-free $\mathbf C [t]$-modules, i.e., of free finitely-generated $\mathbf C [t]$-modules. Taking Euler characteristic, we conclude that $\chi (\pi _* M(X_t)')$ is independent of *t*. Since $M(X_t)'=M(X_t)$ for generic *t*, we conclude that $\chi (\pi _* M(X)) = \chi (\pi _* M(X_t)) + \chi (\pi _* M(X)_{{\mathrm {tor}}})$. Furthermore, $M(X)_{{\mathrm {tor}}}$ is supported on the singular locus, as $M(X)|_{X{\setminus } Z} = \Omega _{X{\setminus } Z}$ is simple. Therefore $M(X)_{{\mathrm {tor}}}$ is the direct sum of delta submodules, so $\pi _* M(X)_{{\mathrm {tor}}}$ is a vector space concentrated in degree zero. We conclude that

8.10 $$\begin{aligned} \chi \left( {\textsf {HP}}^{DR}_*(X)\right) \ge \chi \left( H_{{\textsf {top}}}^{2-*}(X_t)\right) = \chi \left( H_{{\textsf {top}}}^{2-*}(X)\right) +\sum _{s \in Z} \mu _s. \end{aligned}$$

Next, *M*(*X*) has finite length (as it is holonomic) and its composition factors consist of a single copy of ${\text {IC}}(X) := j_{!*} \Omega _{X{\setminus } Z}$, for $j: X{\setminus } Z \rightarrow X$ the open embedding, together with some delta-function D-modules supported on *Z*. Therefore, we also obtain, for ${\text {IH}}^*(X) := \pi _* {\text {IC}}(X)$ the intersection cohomology of *X*,

8.11 $$\begin{aligned} \chi \left( {\textsf {HP}}^{DR}_*(X)\right) = \chi ({\text {IH}}^*(X)) + m, \end{aligned}$$

where *m* is the number of delta-function D-modules appearing as composition factors of *M*(*X*). Putting (8.10) and (8.11) together, we get

8.12 $$\begin{aligned} \chi ({\text {IH}}^*(X)) + m \ge \chi \left( H_{{\textsf {top}}}^{2-*}(X)\right) +\sum _{s \in Z} \mu _s. \end{aligned}$$

Now we restrict again to $X=U_s$, a contractible neighborhood of *s*. As we saw above, in this case $\mu _s$ is the length of the maximal quotient of $M(U_s)$ supported at *s*. Let *K* be the maximal submodule of $M(X)_{\text {ind}}$ supported at *s*. Let $m'$ be the length of *K* (so $K \cong \delta _s^{m'}$). Then, we have an exact sequence $0 \rightarrow K \rightarrow M(X)|_{U_s} \rightarrow \delta ^{\mu _s} \rightarrow 0$, and hence $m=\mu _s+m'$. Since $U_s$ is contractible, we obtain from all of this:

8.13 $$\begin{aligned} \chi ({\text {IH}}^*(U_s)) + m' \ge 1. \end{aligned}$$

Finally we explicitly compute $\chi ({\text {IH}}^*(U_s))$ [25, Proposition 5.9] using the classical formula [28, Sect. 6.1], obtaining $\chi ({\text {IH}}^*(U_s)) = 1 - \dim H_{{\textsf {top}}}^1(U_s {\setminus } \{s\})$. We conclude $m' \ge \dim H_{{\textsf {top}}}^1(U_s {\setminus } \{s\})$. On the other hand, we compute in general that $\dim H_{{\textsf {top}}}^1(U_s {\setminus } \{s\}) = \dim {\text {Ext}}^1({\text {IC}}(U_s), \delta _s)$, the maximum possible size of a submodule of *M*(*X*) supported at *s* which does not contain a direct summand of *M*(*X*). In other words, this is one less than the length of $H^0j_! \Omega _{X{\setminus } Z}$ itself. Therefore, the map $H^0 j_! \Omega _{X{\setminus } Z} \rightarrow M(X)$ is injective, and moreover, (8.13) is an equality. The former finishes the proof of Theorem 8.7, and the latter proves Theorem 8.13.

### Sketch of proof of Theorem 8.9

Here, following [23, Sect. 6], we sketch the proof of Theorem 8.9 under the additional assumption that *f* is actually homogeneous, i.e., $a_i=|x_i|=1$ for all *i*. The general case does not significantly change the details, and this makes things a bit more concrete and easier to follow.

Let ${\text {Eu}}= \sum _i x_i \partial _i$ be the Euler vector field. The main idea is that there is a canonical endomorphism $T_{{\text {Eu}}} \in {\text {End}}_{\mathcal {D}(\mathbf C ^3)}(i_\natural M(X))$ which descends from the endomorphism $\Phi \mapsto {\text {Eu}}\cdot \Phi$ of left multiplication on $\mathcal {D}(\mathbf C ^3)$. (Actually, *M*(*X*) is weakly equivariant, which means that $i_\natural M(X)$ is a graded right $D(\mathbf C ^3)$-module, such that for homogeneous $\Phi \in i_\natural M(X)$, $|\Phi | \Phi = T_{{\text {Eu}}}(\Phi )-\Phi \cdot {\text {Eu}}$; see Sect. 9.) Let $T_{{\text {Eu}}}$ also denote the corresponding endomorphism of *M*(*X*). Then, *M*(*X*) decomposes as a direct sum of the generalized eigenspaces of $T_{{\text {Eu}}}$; call them $M(X)_m$.

Next, the degree in which $M(X)_{\text {ind}}$ appears is the degree *d* of the Poisson bivector $\sigma$, since the action of ${{\text {Eu}}}$ by right multiplication on the symplectic volume form of $X{\setminus }\{0\}$ is multiplication by *d*, and the symplectic volume form is the image of the generator 1 under the canonical quotient $\mathcal {D}_{X{\setminus }\{0\}} \twoheadrightarrow \Omega _{X{\setminus }\{0\}}$ (as it is invariant under Hamiltonian flow). So $M(X)_d$ is a direct sum of $M(X)_{\text {ind}}$ and some $\delta$-function D-modules. By Theorem 8.7, we have an exact sequence

8.14 $$\begin{aligned} 0 \rightarrow H^0 j_! \Omega _{X {\setminus } \{0\}} \rightarrow M(X)_d \rightarrow \delta _0 \otimes {\textsf {HP}}_0(\mathcal {O}(X))_d \rightarrow 0. \end{aligned}$$

Since the Poisson bracket has degree *d* and the only elements of weight zero are constants which are central, $\{\mathcal {O}(X),\mathcal {O}(X)\}$ is spanned by elements of weight $> d$, and hence ${\textsf {HP}}_0(\mathcal {O}(X))_d = \mathcal {O}(X)_d$. In [23, Proposition 3.11] this space is shown to have dimension $g_0$ (using the identification of $H^{\dim X - 1}(D,\mathcal {O}_D)$ with the vector space appearing in [23, Conjecture 3.8] as we remarked in the footnote to Conjecture 8.8). Therefore, Theorem 8.9 reduces to the statement that $M(X)_d$ itself is indecomposable, hence $M(X)_d = M(X)_{\text {ind}}$.

To show this, we first claim that for each $m \in \mathbf Z$, $T_{{\text {Eu}}}$ is actually central in ${\text {End}}(M(X)_m)$. Indeed, every endomorphism of *M*(*X*) descends from left multiplication $T_{\Psi }: \Phi \mapsto \Psi \cdot \Phi$ by some $\Psi \in \mathcal {D}(\mathbf C ^3)$. Since $[T_{{\text {Eu}}},T_{\Psi }] = T_{[{\text {Eu}},\Psi ]}$ and $[{\text {Eu}},-]$ is a semisimple operator on $\mathcal {D}(\mathbf C ^3)$, it follows that $[T_{{\text {Eu}}},-]$ is a semisimple operator on ${\text {End}}(M(X))$. On each generalized eigenspace $M(X)_m$ we therefore have that $[T_{{\text {Eu}}},{\text {End}}(M(X)_m)]=0$.

As a result, $T_{{\text {Eu}}}$ preserves every direct summand of $M(X)_m$ for every *m*. For a contradiction, suppose that there is a direct summand *K* of $M(X)_d$ supported at the origin. We can assume $K \cong \delta _0$. Now $T':=T_{{\text {Eu}}}-d{\text {Id}}$ is a nilpotent endomorphism of $M(X)_d$ which preserves *K*, hence $T'|_K=0$.

To obtain a contradiction, let $v: M(X)_d \rightarrow K \cong \delta _0$ be the projection. Let *N* be the space of smooth distributions on $\mathbf C ^3$ and $w: \delta _0 \rightarrow N$ the inclusion. The main step in the proof is the following (which is a slightly weaker version of [23, Lemma 6.1]):

#### Lemma 8.14

There is a Hamiltonian-invariant distribution $\phi _v \in N$ such that $\phi _v \cdot ({\text {Eu}}-d) = w(v(1))$.

Equivalently, there is a solution $\varphi \in {\text {Hom}}(M(X)_d, N)$, with $\varphi (1)=\phi _v$, such that $\varphi \circ T' = w \circ v$. Since $w \circ v|_{K} \ne 0$, it follows that $T'|_K \ne 0$, which is a contradiction.

To prove Lemma 8.14, we construct $\phi _v$ explicitly. By (8.14) we can associate with *v* an element of ${\textsf {HP}}_0(\mathcal {O}(X))_d^*$. Let $Q \in {\textsf {HP}}_0(\mathcal {O}(X))_d$ be the dual of *v* under the Hermitian pairing on ${\textsf {HP}}_0(\mathcal {O}(X))_d$:

$$\begin{aligned} \langle P,Q \rangle := \int _{S^{5} \cap X}P\overline{Q} \omega \wedge \overline{\omega }/\mathrm{d}r, \end{aligned}$$

where $\omega$ is the rational volume form on *X* (the inverse of the Poisson bivector), and *r* is the radial coordinate function. Then, we partially define $\phi _v$ by:

$$\begin{aligned} \phi _v(\alpha )=-\,2\int _X \alpha \omega \wedge \overline{Q} \overline{\omega }. \end{aligned}$$

This converges when $\alpha$ vanishes to order greater than *d* [23, Lemma 6.2], and we can extend it arbitrarily to a linear functional on all of $C_c^{\infty }(\mathbf C ^3)$. The result is annihilated by all Hamiltonian vector fields which vanish to order greater than *d*, but since the weight of $\sigma$ is *d*, this includes all Hamiltonian vector fields. Thus $\phi _v \in {\text {Hom}}(M(X)_d,N)$.

We claim that $\phi _v \cdot ({\text {Eu}}-d) = w(v(1))$, which completes the proof. By definition of $Q \in {\textsf {HP}}_0(\mathcal {O}(X))_d = \mathcal {O}(X)_d$, the RHS has the property $w(v(1))(PH) = \langle Q,P\rangle H(0)$ for all $P \in \mathcal {O}(X)_d$ and $H \in C_c^\infty (\mathbf C ^n)$. We have to show the same for the LHS. It suffices to show this for a single choice of *H*, and the identity therefore reduces to an explicit computation: choosing $H(x)=h(|x|^2)$ for *h* a smooth real function with $h(0) \ne 0$, we obtain

$$\begin{aligned} (\phi _v \cdot ({\text {Eu}}-d))(PH)= & {} -\,2\int _X P\overline{Q} {\text {Eu}}(H) \omega \wedge \overline{\omega } \\= & {} -\,2\int _0^\infty \mathrm{d}r \cdot r \cdot h'(r^2) \cdot \langle P,Q \rangle \\= & {} \langle P,Q \rangle h(0) = \langle P,Q \rangle H(0). \end{aligned}$$

The first equality uses that we can choose the extension of $\phi _v$ from functions vanishing to order $> d$ at 0 to all functions in such a way that we project away from a space of functions supported in an arbitrarily small neighborhood of zero (and that $\phi _v \cdot ({\text {Eu}}-d)$ does not depend on the choice of extension). This completes the sketch.

### Generalization to locally complete intersections and higher dimension

Finally, we explain how to generalize from surfaces in $\mathbf C ^3$ to general locally complete intersections of arbitrary dimension.

Suppose that *X* is a complete intersection surface in $\mathbf C ^n$, i.e., $X = \{f_1=\cdots =f_{n-2}=0\}$. Then, *X* has a Poisson structure, called the Jacobian Poisson structure, given by the skew-symmetric biderivation $\sigma = i_{\partial _1 \wedge \cdots \wedge \partial _n} (df_1 \wedge \cdots \wedge df_{n-2})|_X$, i.e., $\sigma = \sum _{i<j} \sigma _{i,j} (\partial _i \wedge \partial _j)|_{X}$ for $\sigma _{i,j} = (-1)^{i+j-1}\frac{\partial (f_1,\ldots ,f_{n-2})}{\partial (x_1,\ldots ,\widehat{x_i},\ldots ,\widehat{x_j},\ldots ,x_n)}$ the Jacobian determinant (omitting $x_i$ and $x_j$ from the denominator). Explicitly,

$$\begin{aligned} \{g,h\} = \frac{\partial (f_1,\ldots ,f_{n-2},g,h)}{\partial (x_1,\ldots ,x_n)}. \end{aligned}$$

With this modification all of the preceding results generalize without change, except that now the family $X_t$ should be defined by picking a generic line $\mathbf C \cdot (c_1,\ldots ,c_{n-2}) \subseteq \mathbf C ^{n-2}$, and setting $X_t = \{f_i=c_i\}_{i=1}^n \subseteq \mathbf C ^n$, which will be smooth for generic *t*. The quasi-homogeneous condition is replaced by the condition that *all* of the $f_i$ be quasi-homogeneous with respect to fixed weights $a_i = |x_i|$ (but the degrees of the $f_i$ can be different).

Next, we explain how to generalize to varieties of higher dimension. If $X=\{f_1=\cdots =f_{n-k}=0\} \subseteq \mathbf C ^n$ is a complete intersection of dimension $k \ge 2$, then *X* is equipped with a canonical skew-symmetric multiderivation $\Xi _X: \mathcal {O}(X)^{\otimes \dim X} \rightarrow \mathcal {O}(X)$,

$$\begin{aligned} \Xi _X := i_{\partial _1 \wedge \cdots \wedge \partial _n}(df_1 \wedge \cdots \wedge df_{n-k}) |_{X}. \end{aligned}$$

Given a $(k-2)$-form $\alpha \in \Omega ^{k-2}(X)$, we can associate a vector field, $\xi _\alpha :=i_{\Xi _X}(d\alpha )$, which we call the *Hamiltonian vector field of* $\alpha$. Let $\mathfrak {g}$ be the set of all Hamiltonian vector fields on *X*. We can check that $\mathfrak {g}$ is a Lie algebra, using the formula $[\xi _\alpha , \xi _\beta ] = \xi _{L_{\xi _\alpha } \beta }$ [25, Sect. 3.4], which one can check in local coordinates.

#### Remark 8.15

The vector fields $\xi _\alpha$ actually make sense as vector fields on all of $\mathbf C ^n$: since $\widetilde{\alpha } := df_1 \wedge \cdots \wedge df_{n-k} \wedge \alpha$ is a well-defined $(n-1)$-form on $\mathbf C ^n$, we obtain $\widetilde{\xi _\alpha } := i_{\partial _1 \wedge \cdots \wedge \partial _n}(df_1 \wedge \cdots \wedge df_{n-k} \wedge \alpha )$, a well-defined vector field on $\mathbf C ^n$, which is Hamiltonian on each level set $X_{c_1,\ldots ,c_{n-k}} = \{f_i=c_i\}_{i=1}^{n-k}$. Thus, the Lie algebra of Hamiltonian vector fields on each $X_{c_1,\ldots ,c_{n-k}}$ is the same Lie algebra $\mathfrak {g}$.

Thus, we can replace ${\textsf {HP}}_0(\mathcal {O}(X))$ by $(\mathcal {O}(X))_{\mathfrak {g}}={\textsf {HP}}_0^{\mathfrak {g}-DR}(X)$ and similarly for $X_t$ (where now we define $X_t$ using a generic line $\mathbf C \cdot (c_1,\ldots ,c_{n-k}) \subseteq \mathbf C ^{n-k}$). Similarly we can replace *M*(*X*) and $M(X_t)$ by $M(X,\mathfrak {g})$ and $M(X_t,\mathfrak {g})$. (In view of Remark 8.15, we have the explicit description $(i_t)_\natural M(X_t,\mathfrak {g})=(\mathfrak {g}\mathcal {D}_\mathbf{C ^n} + I_{X_t} \mathcal {D}_\mathbf{C ^n}){\setminus } \mathcal {D}_\mathbf{C ^n}$.) With this change, all of the results and proofs continue to go through in the same manner.

Finally, since the D-module *M*(*X*) is defined locally, we actually do not need to require that *X* globally be a complete intersection, only that it locally admits this structure, provided that there is a global skew-symmetric multiderivation $\Xi _X: \mathcal {O}_X^{\otimes \dim X} \rightarrow \mathcal {O}_X$ which is nonvanishing on the smooth locus. We do not require *X* to be affine. The notion of isolated singularities still makes sense. We can replace the quasi-homogeneous condition by the condition that, at every point $x \in X$, the formal neighborhood ${\hat{X}}_x$ of the point is isomorphic to a complete intersection in ${\hat{\mathbf{C }}}^n_0$ defined by quasi-homogeneous functions (with respect fixed weights that can depend on the point *x* chosen), i.e., that *X* be formally locally conical.

### Relationship to the Bernstein–Sato polynomial

In [14], following a suggestion of the first author, the second author and T. Bitoun relate these results to the theory of the Bernstein–Sato polynomial. Namely, the latter involves the study, for $f \in \mathbf C [x_1,\ldots ,x_n]=\mathcal {O}(\mathbf C ^n)$ and $\lambda \in \mathbf C$, of the left $\mathcal {D}(\mathbf C ^n)$-module $\mathcal {D}(\mathbf C ^n) \cdot f^\lambda$. Let us recall the definition. Let $U := \mathbf C ^n {\setminus } \{f=0\}$, so that $\mathcal {D}(U) = \mathcal {D}(\mathbf C ^n)[f^{-1}]$ and $\mathcal {O}(U) = \mathcal {O}(\mathbf C ^n)[f^{-1}]$. Let $\mathcal {D}(U) \cdot f^\lambda$ be the $\mathcal {D}(U)$-module which as a $\mathcal {O}(U)$-module is the trivial line bundle on *U* together with a nonvanishing section denoted $f^\lambda$, and the $\mathcal {D}(U)$ action is defined by the formula $\xi \cdot f^\lambda = \lambda \xi (f) f^{-1} \cdot f^{\lambda }$ for every derivation $\xi \in {\text {Vect}}(U)$. In other words, $\mathcal {D}(U) \cdot f^\lambda = \mathcal {D}(\mathbf C ^n)[f^{-1}]/\mathcal {D}(\mathbf C ^n)[f^{-1}] \cdot (\partial _i - \lambda f^{-1} f_{x_i} \mid 1 \le i \le n)$ with generator denoted by $f^\lambda$. Let $\mathcal {D}(\mathbf C ^n) \cdot f^\lambda$ be the $\mathcal {D}(\mathbf C ^n)$-submodule of $\mathcal {D}(U) \cdot f^\lambda$ generated by $f^\lambda$. We similarly define the $\mathcal {D}(\mathbf C ^n)[s]$-module $\mathcal {D}(\mathbf C ^n)[s] \cdot f^s$. For every right $\mathcal {D}(\mathbf C ^n)$ module, let $M^{\ell } := M \otimes _{\mathcal {O}(\mathbf C ^n)} \Omega _\mathbf{C ^n}^{-1}$ be the associated left $\mathcal {D}(\mathbf C ^n)$-module. For $X = \{f=0\}$, one has the following canonical surjection of left $\mathcal {D}(\mathbf C ^n)$-modules:

8.15 $$\begin{aligned} i_\natural M(X,\mathfrak {g})^\ell= & {} i_\natural M(X,\mathfrak {g}) \otimes _{\mathcal {O}(\mathbf C ^n)} \Omega _\mathbf{C ^n}^{-1} \twoheadrightarrow \mathcal {D}(\mathbf C ^n) f^\lambda / \mathcal {D}(\mathbf C ^n) f^{\lambda +1}, \nonumber \\&1 \otimes (\partial _1 \wedge \cdots \wedge \partial _n) \mapsto f^\lambda , \end{aligned}$$

as well as a canonical map

8.16 $$\begin{aligned} i_\natural M(X,\mathfrak {g})^\ell \rightarrow \mathcal {D}(\mathbf C ^n)[s] f^s / \mathcal {D}(\mathbf C ^n)[s] f^{s+1}, \end{aligned}$$

which is surjective for *f* quasi-homogeneous. (Since then for ${\text {Eu}}$ the Euler vector field, we have $|f|^{-1} {\text {Eu}}\cdot f^s = sf^s$.) Using the maps (8.15) and (8.16), we prove the following. Let $Z \subseteq X$ continue to be the singular locus, which we assume to be finite. For each $s \in Z$, let $U_s$ be a contractible neighborhood of *s*. Define

$$\begin{aligned} \begin{aligned} K&:= \ker (H^0 j_!\Omega _{X{\setminus } Z} \twoheadrightarrow j_{!*} \Omega _{X{\setminus } Z}) \cong \bigoplus _{s \in Z} {\text {Ext}}^1(j_{!*} \Omega _{X{\setminus } Z}, \delta _s)^* \otimes \delta _s \\&\cong \bigoplus _{s \in Z} H_{{\textsf {top}}}^{n -2} (U_s {\setminus } \{s\})^* \otimes \delta _s \end{aligned} \end{aligned}$$

(for the last isomorphism see [14, Lemma 3.5], cf. Example 8.10). Then:

#### Theorem 8.16

[14] Suppose that $n \ge 3$ and $\{f=0\}$ has isolated singularities. Then, (8.15) produces an isomorphism $(i_\natural M(X,\mathfrak {g})_{\text {ind}}/K)^\ell \cong \mathcal {D}(\mathbf C ^n) f^{-1} / \mathcal {O}(\mathbf C ^n)$. If moreover *f* is quasi-homogeneous, (8.16) produces an isomorphism $i_\natural M(X,\mathfrak {g})^\ell \cong \mathcal {D}(\mathbf C ^n)[s] f^s / \mathcal {D}(\mathbf C ^n)[s] f^{s+1}$.

#### Corollary 8.17

Conjecture 8.8 is equivalent to the statement: if $\{f=0\}$ is irreducible with isolated singularities, then the length of the left $\mathcal {D}(\mathbf C ^n)$-module $\mathcal {D}(\mathbf C ^{n}) \cdot f^{-1} / \mathcal {O}(\mathbf C ^n)$ is one more than the sum of the reduced genera of the singularities.

This formula was motivated by a similar phenomenon discovered by T. Bitoun in characteristic $p > 0$: Let *f* be a polynomial with rational coefficients in $n \ge 3$ variables which is absolutely irreducible, i.e., it remains irreducible over $\mathbf C$, and assume that $\{f=0\}$ has a unique singular point. For a field *F* let $\mathbf A _F^n$ be the affine space over the field *F* and $\mathcal {D}(\mathbf A _F^n)$ be the ring of Grothendieck differential operators on $\mathbf A _F^n$. Then, for *p* sufficiently large, we can consider the local cohomology $\mathcal {D}(\mathbf A _\mathbf{F _p}^n)$-module $\mathcal {O}(\mathbf A _\mathbf{F _p}^n)[f^{-1}] / \mathcal {O}(\mathbf A _\mathbf{F _p}^n)$, which turns out to be generated by $f^{-1}$ (hence fully analogous to $\mathcal {D}(\mathbf C ^n) \cdot f^{-1} / \mathcal {O}(\mathbf C ^n)$ in characteristic zero). In [9], it is proved that, for sufficiently large *p* the length of this $\mathcal {D}(\mathbf A _\mathbf{F _p}^n)$-module is one more than the “*p*-genus” of the singularity, which is defined as the dimension of the “stable part” of $H^{n-2}(Y_{\overline{p}}, \mathcal {O}_{Y_{\overline{p}}})$, for $Y_{\overline{p}}$ the exceptional fiber of a resolution of singularities of $X_{\overline{p}}:=\{f=0\} \subseteq \mathbf A _{\overline{\mathbf{F _p}}}$, where the stable part refers to the intersection of the images of all powers of the Frobenius morphism. See [9, 14] for details.

The above results also have a direct application to the Bernstein–Sato polynomial. Recall that the Bernstein–Sato polynomial is defined as the annihilator in $\mathbf C [s]$ of $\mathcal {D}(\mathbf C ^n)[s] f^s / \mathcal {D}(\mathbf C ^n)[s] f^{s+1}$. Using the surjections $\mathcal {D}(\mathbf C ^n)[s] f^s \twoheadrightarrow \mathcal {D}(\mathbf C ^n) \cdot f^\lambda$, it follows that, if $\mathcal {D}(\mathbf C ^n) \cdot f^\lambda \ne \mathcal {D}(\mathbf C ^n) \cdot f^{\lambda +1}$, then $\lambda$ is a root of this polynomial. The converse question is interesting and little is known aside from [43, Proposition 6.2], which says that the converse holds if additionally $\lambda -m$ is not a root for any positive integer *m*. The results above imply a converse in the quasi-homogeneous case:

#### Corollary 8.18

[14, 52] For *f* quasi-homogeneous with an isolated singularity and $\lambda \in \mathbf C$, if $\lambda$ is a root of the Bernstein–Sato polynomial, then $\mathcal {D}(\mathbf C ^n) \cdot f^\lambda \ne \mathcal {D}(\mathbf C ^n) \cdot f^{\lambda +1}$.

M. Saito has also demonstrated in [52, Example 4.2] a counterexample to this assertion when the quasi-homogeneity assumption is removed.

## Weights on homology of cones

We continue to work over $\mathbf k =\mathbf C$. In Sect. 8.4, we used that if $\mathcal {O}(X)$ is graded (i.e., *X* admits a $\mathbf C ^\times$-action) and Poisson with a homogeneous Poisson bracket (which was the case there for *X* a quasi-homogeneous surface in $\mathbf C ^3$), then ${\textsf {HP}}_0(\mathcal {O}(X))$ is also nonnegatively graded. In this section we explain how in fact ${\textsf {HP}}_*^{DR}(X)$ is bigraded, and consider its bigraded Hilbert–Poincaré polynomial (i.e., a Laurent polynomial in two variables). We give several examples (without proofs) that demonstrate that one recovers in this way many special polynomials from combinatorics.

### Weakly equivariant D-modules and bigrading on Poisson-de rham homology

As noted in Sect. 8.4 in a particular situation, when $\mathcal {O}(X)$ is graded, then *M*(*X*) is weakly $\mathbf C ^\times$-equivariant. Let us first recall the general definition:

#### Definition 9.1

Let $\mathcal {O}(X)$ be graded and $i: X \rightarrow V$ a $\mathbf C ^\times$-equivariant embedding into a smooth variety *V*, i.e., $\mathcal {O}(V)$ is graded and $I_X$ is a graded ideal. Then, a weakly $\mathbf C ^\times$-equivariant D-module *M* on *X* is one associated with a graded right $\mathcal {D}(V)$-module $i_\natural M$ on *V* supported on *i*(*X*).

Call this grading on $i_\natural M(X)$ the *weight grading*. In this case, the pushforward $\pi _* M(X) = i_\natural M(X) \otimes ^L_{\mathcal {D}(V)} \mathcal {O}(V)$ under the map $\pi : X \rightarrow \text {pt}$ is a complex of weight graded vector spaces, since $\mathcal {O}(V)$ is also a graded $\mathcal {D}(V)$-module. Therefore, the Poisson-de Rham homology ${\textsf {HP}}^{DR}_*(X)$ is bigraded. It is very interesting to compute this grading, which turns out to be closely related to important special polynomials.

#### Remark 9.2

The weight grading on *M*(*X*) comes from a decomposition of *M*(*X*) itself. Let ${\text {Eu}}_X$ and ${\text {Eu}}_V$ be the Euler vector fields on *X* and *V*, i.e., ${\text {Eu}}_X(f) = |f| f$ when $f \in \mathcal {O}(X)$ is homogeneous and similarly for *V*. Then, the endomorphism $\Phi \mapsto {\text {Eu}}_V \cdot \Phi$ of $\mathcal {D}(V)$ induces an endomorphism of *M*(*X*), which can also be described as (i) the endomorphism induced by $\Phi \mapsto {\text {Eu}}_X \Phi$ on the D-module $\mathcal {D}_X$ on *X*, and (ii) the endomorphism of $i_\natural M$ which on homogeneous elements is $\Phi \mapsto |\Phi | \Phi - \Phi \cdot {\text {Eu}}_V$. Call this endomorphism $T_{{\text {Eu}}}$. Then, *M*(*X*) decomposes into its generalized eigenspaces under $T_{{\text {Eu}}}$, $M(X) = \bigoplus _{m \in \mathbf Z } M(X)_m$, and $\pi _* M(X)_m$ is concentrated in weight *m*. This decomposition is important to the proof of the following facts, as well as yielding interesting information in its own right. (It allows one to define, for instance, canonical filtrations on irreducible representations of the Weyl group: see Sect. 9.3 below.)

### Symmetric powers of conical surfaces with isolated singularities

Let $Y \subseteq \mathbf C ^3={\textsf {Spec}}[x_1,x_2,x_3]$ be a conical surface with singular locus $\{0\}$, i.e., $Y = \{f=0\}$ where *f* is quasi-homogeneous with isolated singularities. In this context, $\bigoplus _{n \ge 0} {\textsf {HP}}_0(\mathcal {O}(S^n Y))^*$ is again a graded algebra just as in Sect. 7.1, with grading given by *n*, which we call the “symmetric power.” In fact, it is a bigraded algebra, with the additional grading given by the weight on each summand ${\textsf {HP}}_0(\mathcal {O}(S^n Y))^*$ (which will be nonpositive, as it is dual to the nonnegative weight on $\mathcal {O}(S^n Y)$). It turns out to be possible to explicitly compute this bigraded vector space:

#### Theorem 9.3

[22, Theorem 1.1.14] There is a (noncanonical) isomorphism of bigraded algebras,

9.1 $$\begin{aligned} {\textsf {Sym}}({\textsf {HP}}_0(\mathcal {O}(Y))^*[t]) \cong \bigoplus _{n \ge 0} {\textsf {HP}}_0(\mathcal {O}(S^n Y))^*, \end{aligned}$$

assigning *t* bidegree $(1,-|f|)$ (with 1 the symmetric power and $-|f|$ the weight), and ${\textsf {HP}}_0(\mathcal {O}(S^n Y))^*$ is assigned symmetric power *n* (on both sides of the equation).

Let $J_Y := \mathcal {O}(\mathbf C ^3) / (f_{x_1},f_{x_2},f_{x_3})$ be the Jacobi ring (so $\dim J_Y = \mu _Y$ is the Milnor number of the singularity at the origin), and let its Hilbert–Poincaré polynomial be $h(J_Y;t) = \sum _{i=1}^{\mu _Y} t^{n_i}$. Note that for $Y = \mathbf C ^2/\Gamma$ with $\Gamma < \textsf {SL}(2,\mathbf C )$ finite, it is well known that $n_i = d_i-2$ for $d_i$ the degrees of the fundamental invariants of the Weyl group attached to $\Gamma$ by the McKay correspondence (see (8.1 8.2 8.3 8.4)), i.e., $\mathbf C [\mathfrak {h}]^W$ is a polynomial algebra generated by elements of degrees $d_i$ with $\mathfrak {h}$ the reflection representation of *W* and $|\mathfrak {h}^*|=1$.

#### Corollary 9.4

[22, (1.1.16)] $\displaystyle \sum _{n \ge 0} h({\textsf {HP}}_0(\mathcal {O}(S^n Y))^*;t) s^n = \prod _{i=1}^{\mu _Y} \prod _{j \ge 0} \frac{1}{1-t^{n_i + jd}s^{j+1}}$. In particular, the dimension of ${\textsf {HP}}_0(\mathcal {O}(S^n Y))$ is $a_n(\mu _Y)$ (7.3), the number of $\mu _Y$-multipartitions of *n*.

#### Remark 9.5

We put “noncanonical” in parentheses because, unlike in the case of (7.1), we do not construct an explicit isomorphism. Note that the map of (7.1) still makes sense here, but is no longer injective: indeed, if $\varepsilon _n: \mathcal {O}(S^n Y) \rightarrow \mathbf C$ is the augmentation map, then the map of (7.1) sends $\varepsilon _1 t^{m-1}$ to $\varepsilon _m$ for all *m*, but $\varepsilon _{m_1} \cdots \varepsilon _{m_k} = \varepsilon _{m_1+\cdots +m_k}$ in the RHS, which shows that $\varepsilon _1 \& \varepsilon _1$ and $\varepsilon _1 t$ both map to $\varepsilon _2$. It does not appear that there exists a canonical isomorphism in general.

Now let us restrict to the special case where $Y \cong \mathbf C ^2/\Gamma$ for $\Gamma < \textsf {SL}(2,\mathbf C )$ finite, i.e., *Y* is a du Val singularity. Then, $S^n Y = \mathbf C ^{2n}/(\Gamma ^n \rtimes S_n)$ is a finite linear quotient of a vector space, so the special case of (4.4) with *V* linear (i.e., [20, Proposition 4.16]), yields the entire structure of $M(S^n Y)$ for every *n*. We can therefore compute the entire Poisson-de Rham homology, with the help of [50, Theorem 5.1], which tells us in this case that a slight modification of (4.4) is an isomorphism of weakly $\mathbf C ^\times$-equivariant D-modules, applying on the right-hand side a shift in weight down by $\dim Z$ in each summand. The result is:

#### Corollary 9.6

For $Y=\mathbf C ^2/\Gamma$, there is a (noncanonical) isomorphism of trigraded algebras,

9.2 $$\begin{aligned} {\textsf {Sym}}({\textsf {HP}}_0(\mathcal {O}(Y))^*[t] \oplus \mathbf C [s] \cdot u) \cong \bigoplus _{n \ge 0} {\textsf {HP}}_*^{DR}(\mathcal {O}(S^n Y))^*, \end{aligned}$$

where in the trigrading by symmetric power, weight, and homological degree, $|t|=(1,-|f|,0)$, $|s|=(1,0,2)$, and $|u|=(1,-2,2)$. Thus on trigraded Hilbert series we obtain:

9.3 $$\begin{aligned} \begin{aligned}&\sum _{n,m \ge 0} h\left( {\textsf {HP}}_m^{DR}(\mathcal {O}(S^n Y))^*;t\right) s^nu^m = \prod _{j \ge 0} \frac{1}{1-s^{j+1} t^{-2}u^{2j}}\prod _{i=1}^{\mu _Y} \prod _{j \ge 0} \frac{1}{1-t^{n_i + jd}s^{j+1}}. \end{aligned} \end{aligned}$$

In particular $\dim {\textsf {HP}}^{DR}_*(S^n Y) = a_n(\mu _Y+1)$, the number of $(\mu _Y+1)$-multipartitions of *n* (where $\mu _Y$ also equals the number of irreducible representations of $\Gamma$).

### The Nilpotent cone

Next let $X \subseteq \mathfrak {g}$ be the cone of nilpotent elements in a semisimple Lie algebra $\mathfrak {g}$. Then, as explained in Example 6.6, ${\textsf {HP}}_*^{DR}(X) \cong H^{\dim X - *}(T^* \mathcal {B})$, for $T^* \mathcal {B} \rightarrow X$ the Springer resolution. This is isomorphic to the cohomology of the flag variety $\mathcal {B}$ itself. However, the source ${\textsf {HP}}_*^{DR}(X)$ has an additional grading given from the dilation action on *X*. G. Lusztig proposed a formula for the graded Hilbert–Poincaré polynomial [50, Conjecture 8.1]. For *W* the Weyl group attached to $\mathfrak {g}$, and $\chi$ an irreducible representation of *W*, let $K_{\mathfrak {g},\chi }(t)$ be the generalized Kostka polynomial, $K_{\mathfrak {g},\chi }(t) := \sum _{i \ge 0} t^i\dim {\text {Hom}}_W(\chi , H^{2\dim \mathcal {B}- 2i}(\mathcal {B}))$. Then, G. Lusztig’s suggestion was:

9.4 $$\begin{aligned} h\left( {\textsf {HP}}_*^{DR}(X);x,y\right) =\sum _{\chi \in \text {Irrep}(W)} K_{\mathfrak {g},\chi }(x^2) K_{\mathfrak {g},\chi }(y^{-2}). \end{aligned}$$

This is now a theorem [13, 1.1], again making use of [34].

Note that the fact that $H^{2 \dim \mathcal {B}-*}(\mathcal {B})$ has a *W*-action and that ${\textsf {HP}}^{DR}_*(X)$ has a second grading means that the isomorphism ${\textsf {HP}}^{DR}_*(X) \cong H^{2 \dim \mathcal {B}-*}(\mathcal {B})$ cannot be canonical (indeed, $K_{\mathfrak {g},\chi }(y^{-2})$ would have to be a multiple of $\dim \chi$ if there were a bigrading compatible with the *W* action, and this is false in general). Instead, in [13], the authors construct a *canonical family of filtrations* on $H^{2 \dim \mathcal {B}-*}(\mathcal {B})$, parameterized by $\lambda \in \mathfrak {h}^*_{{{\text {reg}}}}$, with $\mathfrak {h}$ a Cartan subalgebra of $\mathfrak {g}$ and $\mathfrak {h}^*_{{{\text {reg}}}}$ the complement of the coroot hyperplanes.

#### Theorem 9.7

[13, Theorem 1.3] For every element $\lambda \in \mathfrak {h}_{{\text {reg}}}^*$, there is a canonical associated filtration $\mathcal {F}_\lambda$ on $H^{2 \dim \mathcal {B}-*}(\mathcal {B})$ whose associated graded vector space is ${\textsf {HP}}_*^{DR}(X)$. This is *W*-equivariant: $\mathcal {F}_{w(\lambda )} = w(\mathcal {F}_\lambda )$.

#### Corollary 9.8

[13, Corollary 1.4] On every irreducible representation $\chi$ of *W*, to every element $\lambda \in \mathfrak {h}_{{\text {reg}}}^*$ is associated a canonical filtration whose Hilbert series is $K_{\mathfrak {g},\chi }(y^{-2})$.

#### Example 9.9

[13, Example 1.5] Let $\mathfrak {g}=\mathfrak {sl}_n$ and let $\chi = \mathfrak {h}^* \otimes \text {sign} \cong \mathbf C ^{n-1}$. Consider $\chi$ to be (in coordinates) $\mathbf C ^n / \mathbf C \cdot (1,1,\ldots ,1)$, and make the same identification for $\mathfrak {h}^*$. Let $\lambda \in \mathfrak {h}_{{{\text {reg}}}}^*$ be the image of $(a_1, \ldots , a_n) \in \mathbf C ^n$. Then, the resulting filtration on $\chi$ (the Vandermonde filtration) is: $F^{2i-2 \,\mathrm{dim}\mathcal {B}}(\chi )$ is the span of $(a_1^j, \ldots , a_n^j)$ for $j \le i$.

Finally, we can apply this to the classical W-algebras (see Example 6.7). This uses the Springer correspondence, which associates with every irreducible representation $\chi$ of *W* a pair of a nilpotent coadjoint orbit $O_\chi \subseteq \mathfrak {g}^*$ and a local system $L_\chi$ on $O_\chi$. Identify $\mathfrak {g}^* \cong \mathfrak {g}$ using the Killing form, so that $O_\chi \subseteq \mathfrak {g}$, which allows us to consider irreducible representations $\chi$ such that $O_\chi = G \cdot e$.

#### Corollary 9.10

[13, Corollary 2.5] The Hilbert–Poincaré polynomial of ${\textsf {HP}}_0(\mathcal {O}(S_e^0))$ is

$$\begin{aligned} y^{\dim G \cdot e} \sum _{\chi \in \text {Irrep}(W) \mid O_\chi =G \cdot e} \dim L_\chi \cdot K_{\mathfrak {g},\chi }(y^{-2}). \end{aligned}$$

### The hypertoric case

Let *X* be a hypertoric cone as in Example 6.9, which admits a symplectic resolution. Let $\mathcal {A}$ be the associated hyperplane arrangement (in $\mathbf C ^{\dim X/2}$) with $|\mathcal {A}|$ linear hyperplanes (whose normal vectors span $\mathbf C ^{\dim X/2}$). Let $\Phi _{\mathcal {A}}(x,y,b) := \Phi _{\mathcal {A}}(x,y,b_1,\ldots ,b_{\mathcal {A}})$ be the polynomial defined by G. Denham in [16] using the combinatorial Laplacian.

#### Theorem 9.11

[50, Theorem 6.1] $$h({\textsf {HP}}_*^{DR}(X);x,y) = y^{-\dim X} \Phi _{\mathcal {A}}(x^2-1,y^{-2}-1,y^2)$$.

The above formula is proved via the Tutte polynomial and symplectic leaves, which are interesting in their own right. Let $T_{\mathcal {A}}(x,y)$ denote the Tutte polynomial of the arrangement. The symplectic leaves of *X* are indexed by (coloop-free) flats $F \subseteq \mathcal {A}$, cf. e.g., [51]. For each such we can define the restriction, $\mathcal {A}^F$, by intersecting with all the hyperplanes in *F*, and the localization, $\mathcal {A}_F$, by dividing by the intersection of these hyperplanes. Then, the above formula follows from the following one:

$$\begin{aligned} h\left( {\textsf {HP}}_*^{DR}(X);x,y\right) = y^{-\dim X} \sum _F T_{\mathcal {A}^F}(x^2,0) T_{\mathcal {A}_F}(0,y^{-2}) y^{2|F|}. \end{aligned}$$

### Conjectural description for conical symplectic resolutions

Finally, we sketch a conjectural description of the bigrading on ${\textsf {HP}}^{DR}_*(X)$ in terms of a deformation of resolution (which enhances Conjecture 6.1(b) for conical symplectic resolutions to incorporate the weights). Let $\rho : \widetilde{X} \rightarrow X$ be a projective conical symplectic resolution. Let $\widetilde{\mathcal {X}} \rightarrow \mathcal {X}$ be a $\mathbf C ^{\times }$-equivariant twistor deformation of $\rho$ over $\mathbf C = {\textsf {Spec}}\,\mathbf C [t]$ (see Remark 6.8). Note that the degree of *t* equals the degree of the symplectic form on $\widetilde{X}$; call this degree *d*.

Let ${\widetilde{\theta }}: \widetilde{\mathcal {X}} \rightarrow \mathbf C$ and $\theta : \mathcal {X} \rightarrow \mathbf C$ be the projections to the base, and let $\mathcal {X}_t := \theta ^{-1}(t)$ and $\widetilde{\mathcal {X}}_t := \widetilde{\theta }^{-1}(t)$ be the fibers. By the argument of [56, Sect. 4.2], ${\widetilde{\theta }}$ is a topological fiber bundle (thanks to Y. Namikawa for pointing this out). Thus, the family of vector spaces $H^i(\widetilde{\mathcal {X}}_t)$ is an algebraic vector bundle on $\mathbf C$ for each *i* equipped with the Gauss–Manin connection (corresponding to the right $\mathcal {D}$-module $H^{i-\dim X} {\widetilde{\theta }}_* \Omega _{\widetilde{{\mathcal {X}}}}$). Using this connection we can uniquely trivialize the vector bundle (up to an overall scaling) and identify it with $H^i({\widetilde{X}}) \otimes \mathbf C [t]$. (Note that the family of varieties $\widetilde{\mathcal {X}}$ is nontrivial, since $\widetilde{\mathcal {X}}_t$ is affine for generic *t* but not for $t=0$.)

Let $\Omega _{\text {cl}}^i$ be the space of holomorphic fiberwise closed differential forms on $\widetilde{\mathcal {X}}$, i.e., cycles in the complex of holomorphic differential forms modulo *dt*, for *t* the function $\widetilde{\mathcal {X}} \rightarrow \mathbf C$ above. There is a natural map

9.5 $$\begin{aligned} \Phi : \Omega _{\text {cl}}^i \rightarrow H^i(\widetilde{X}) \otimes \mathbf C [t]. \end{aligned}$$

Let $K_i := {\text {coker}}(\Phi )$. Then, $K_i$ is finite dimensional and concentrated at $t=0$. Thus, as a $\mathbf C [t]$-module, $K_i$ is a direct sum of Jordan blocks $\mathbf C [t]/t^{\phi (i,j)}$, for $j=1, 2, \ldots , \dim (H^i({\widetilde{X}}))$ and $\phi (i,j) \in \mathbf Z _{\ge 0}$ (where some of the Jordan blocks are allowed to be zero, in which case we set $\phi (i,j)= 0$). Recall that *d* is the degree of the generic symplectic form on *X*, which equals the degree of *t*.

#### Conjecture 9.12

$$h({\textsf {HP}}_*^{DR}(X); x,y) = y^{-d \cdot \dim X/2} \sum _{i,j} x^{i} y^{d \cdot \phi (\dim X - i,j)}$$.

The cohomological degree zero case, i.e., $h({\textsf {HP}}_0^{DR}(X);y) = y^{-d \cdot \dim X/2} \sum _j y^{d \cdot \phi (\dim X,j)}$, should follow from Conjecture 6.1(a) using the direct interpretation of ${\textsf {HP}}_0^{DR}(X)\cong {\textsf {HP}}_0(\mathcal {O}(X))$ via functions on *X*. The difficulty appears to be in relating ${\textsf {HP}}_i^{DR}(X)$ for $i> 0$ to fiberwise closed differential forms on the family.

#### Remark 9.13

Note that the conjecture predicts in particular that ${\textsf {HP}}_*^{DR}(X)$ lies only in weights which are multiples of *d*. This is not true in general if *X* does not admit a symplectic resolution, even if it is a symplectic singularity (and hence has finitely many leaves): in [18, Appendix A], many examples are constructed of $X=V/G$ with *V* a symplectic vector space and $G < \textsf {Sp}(V)$ finite, such that ${\textsf {HP}}_0(\mathcal {O}(X))$ is nonzero in degree three (the smallest dimension of *V* in these examples is 12). Since the Poisson bracket on *V* / *G* is the one coming from *V*, having degree $-2$, in this case we have $d=2$ even though the weights are not all even.

We explain how to verify the conjecture in the case of a du Val singularity $X \cong \mathbf C /\Gamma$ for $\Gamma < \textsf {SL}(2,\mathbf C )$ finite. Then, ${\textsf {HP}}_*^{DR}(X)$ is only nonzero in degrees zero and two, and ${\textsf {HP}}_2^{DR}(X) = \mathbf C$ occurring in weight $-d$, by (4.2) together with [50, Theorem 5.1] (note that $H^0({\widetilde{X}})$ is spanned by the constant 1, so $\phi (0,1)=0$). Thus, we only have to check the conjectural formula in degree zero. Let $X = \{f=0\}$ where *f* is the corresponding equation listed in Example 8.3. Let the family be $\mathcal {X} = \{f-t^{h}\} \subseteq \mathbf C ^4={\textsf {Spec}}[x_1,x_2,x_3,t]$, where *h* is the Coxeter number associated with the Dynkin diagram listed in the example (note that $h|t|=|f|$); this can be seen to be a twistor deformation. Let $\omega = \mathrm{d}x_1 \wedge \mathrm{d}x_2 \wedge \mathrm{d}x_3 / df$ be the fiberwise generic symplectic form on $\mathcal {X}$ (constant in *t*), and $\widetilde{\omega }$ be the fiberwise symplectic form on $\widetilde{\mathcal {X}}$, which generically is the pullback $\rho ^*\omega$. Then, we have isomorphisms ${\textsf {HP}}_0(X) \rightarrow H^2(X_t)$ for $t \ne 0$ given by $[g] \mapsto [g \omega ]$. If *g* is homogeneous, then $g \omega$ is a homogeneous form which has degree $|g|+|\omega |=|g|+d$. Therefore, the order of vanishing of $[g \widetilde{\omega }]$ in cohomology at $t=0$ must be precisely $|g|/d+1$. It follows that a homogeneous basis of ${\textsf {HP}}_0(\mathcal {O}(X))$ produces forms $[g \widetilde{\omega }]$ which restrict on each fiber $t \ne 0$ to a basis of the cohomology and which vanish on cohomology at $t=0$ to order precisely $|g|/d+1$. This is exactly what is required for the formula to hold. To complete the proof we have only to show that the elements $g \widetilde{\omega }$ span all fiberwise (closed) two-forms on $\widetilde{\mathcal {X}}$ modulo fiberwise exact forms. To see this, observe that all fiberwise two-forms on $\widetilde{\mathcal {X}}$ are of the form $g \widetilde{\omega }$ for some $g \in \Gamma (\widetilde{\mathcal {X}},\mathcal {O}_{\widetilde{\mathcal {X}}}) = \mathcal {O}(\mathcal {X})$, and that they are fiberwise exact if and only if $g \in \{\mathcal {O}(X),\mathcal {O}(X)\} \otimes _\mathbf{C } \mathbf C [t] \subseteq \mathcal {O}(X) \otimes _\mathbf{C } \mathbf C [t] \cong \mathcal {O}(\mathcal {X})$.

## Appendix A. Background on D-modules

In this appendix, we recall without proofs one way to define the D-modules we need, via Kashiwara’s equivalence (for a reference, see, e.g., [36, 42]; another approach, via crystals, can be found in [3, 30]). Then, we recall the definition of holonomic D-modules and the theorem that they are preserved by direct and inverse image.

### Definition of D-modules on singular varieties

#### Definition A.1

Let *V* be a smooth affine variety. Then, a right D-module on *V* is a right module for the ring $\mathcal {D}(V)$ of differential operators on *V* with polynomial coefficients. If *V* is not necessarily affine, but still smooth, then a right D-module is defined to be a sheaf of right modules over the sheaf $\mathcal {D}_V$ of rings of differential operators. A D-module is called quasi-coherent if the underlying $\mathcal {O}_V$-module is quasi-coherent. Let $\text {mod}-\mathcal {D}_V$ denote the category of quasi-coherent right D-modules on *V*.

#### Definition A.2

Given a closed subset $X \subseteq V$ of a smooth variety *V* with ideal sheaf $\mathcal {I}_X$, and a right D-module *M* on *V*, we say that *V* is supported on *X* if, for every open affine subset $U \subseteq V$ and all local sections $s \in \Gamma (U, M)$ and $f \in \Gamma (U,\mathcal {I}_X)$, there exists $N \ge 1$ such that $s \cdot f^N = 0$.

We caution that the above notion of support, which takes place on *V*, is completely different from the notion of characteristic variety (singular support) which we will use later, which takes place on $T^*V$.

#### Definition A.3

Suppose *X* is an arbitrary (not necessarily smooth or affine) variety equipped with a closed embedding $i: X \rightarrow V$ into a smooth variety *V*. Then, a right D-module on *X* with respect to *i*, is defined to be a right D-module on *V* which is supported on *i*(*X*). It is quasi-coherent if the D-module on *V* is quasi-coherent.

#### Remark A.4

In fact, not every variety admits a closed embedding into a smooth variety, so this definition cannot be used to define D-modules on arbitrary varieties.

In the case that *X* is affine, this definition does not depend on the choice of closed embedding up to canonical equivalence because of the following theorem of Kashiwara. Given a closed embedding $i: Z \rightarrow V$ of smooth varieties, let $\text {mod}_Z-\mathcal {D}_V$ denote the category of right $\mathcal {D}_V$-modules supported on *i*(*Z*). Then, there are functors $i_{\natural }: \text {mod}-\mathcal {D}_Z \rightarrow \text {mod}_Z-\mathcal {D}_V$ and $i^{\natural }: \text {mod}_Z-\mathcal {D}_V \rightarrow \text {mod}-\mathcal {D}_Z$, given by, for $i_\bullet$ and $i^\bullet$ the direct and inverse image of sheaves of vector spaces,

A.1 $$\begin{aligned} i_{\natural }(M)&= i_\bullet (M \otimes _{\mathcal {D}_Z} (\mathcal {O}_Z \otimes _{i^\bullet \mathcal {O}_V} i^\bullet \mathcal {D}_V)), \end{aligned}$$

A.2 $$\begin{aligned} i^{\natural }(M)&:= {\text {Hom}}_{i^\bullet \mathcal {O}_V}(\mathcal {O}_Z, i^\bullet M), \end{aligned}$$

with canonical right D-module structures.

#### Theorem A.5

Suppose that $i: Z \rightarrow V$ is a closed embedding of smooth varieties. Then, the functors $i_{\natural }$ and $i^{\natural }$ above are mutually quasi-inverse equivalences.

#### Theorem A.6

Let *X* be an arbitrary variety. Suppose that $i_1: X \rightarrow V_1$ and $i_2: X \rightarrow V_2$ are two closed embeddings with $V_1, V_2$ smooth, and that there exists a third smooth variety $V_3$ together with a commuting diagram of closed embeddings,

Then, the functors $i_{23}^{\natural } \circ (i_{13})_{\natural }$ and $i_{13}^{\natural } \circ (i_{23})_{\natural }$ define mutually inverse equivalences between the categories of quasi-coherent right D-modules on *X* with respect to $i_1$ and $i_2$. Moreover, this does not depend on the choice of $i_3$ and $V_3$, and the composition of the equivalences from $i_1$ to $i_2$ to $i_3$ and back to $i_1$ is the identity functor.

In the case that *X* is an affine variety, there always exists an embedding $X \rightarrow V$ into an affine space, and given two such embeddings $X \rightarrow V_1$ and $X \rightarrow V_2$ we can always find a third affine space $V_3$ such that we obtain a commuting diagram of embeddings as above. Therefore we conclude:

#### Corollary A.7

If *X* is an affine variety, there is a category $\mathcal {D}-\text {mod}_X$ of quasi-coherent D-modules on *X* which is canonically equivalent to the category of quasi-coherent right D-modules on *V* supported on *i*(*X*) for every choice of closed embedding $i: X \rightarrow V$ with *V* an affine space (or a smooth affine variety).

Gluing these categories together by Theorem A.6, we obtain a canonical category on general varieties:

#### Corollary A.8

For a general variety *X*, there is a canonical abelian category $\mathcal {D}-\text {mod}_X$ of quasi-coherent D-modules on *X* such that for every open affine subset $U \subseteq X$, there is a canonical exact restriction functor $\mathcal {D}-\text {mod}_X\rightarrow \mathcal {D}-\text {mod}_{U}$.

#### Corollary A.9

Given any embedding $i: X \hookrightarrow V$ of a variety *X* into a smooth variety *V*, there are canonical equivalences $i_{\natural }: \mathcal {D}-\text {mod}_X\rightarrow \text {mod}_X-\mathcal {D}_V$ and $i^{\natural }: \text {mod}_X-\mathcal {D}_V \rightarrow \mathcal {D}-\text {mod}_X$.

#### Remark A.10

On a singular variety *X* we refer only to D-modules, without specifying right or left, for the following reason. The category $\mathcal {D}-\text {mod}_X$ of D-modules on *X* is abstractly defined only up to canonical equivalence and in general does not identify with modules over any sheaf of rings on *X* itself. Since the categories of left and right D-modules on a smooth variety *V* are themselves canonically equivalent (via the functors $M \leftrightarrow M \otimes _{\mathcal {O}_V} \Omega _V$ for *M* a left D-module and $M \otimes _{\mathcal {O}_V} \Omega _V$ the corresponding right D-module), using either one yields the same definition of the category $\mathcal {D}-\text {mod}_X$. The objects of this canonical category $\mathcal {D}-\text {mod}_X$ can be thought of equivalently either as local collections of left D-modules or of right D-modules on embeddings of open subsets of *X* into smooth varieties.

### The D-module $\mathcal {D}_X$

There is a global sections functor for D-modules on singular varieties which is defined as follows. In the case *X* is equipped with a closed embedding $i: X \rightarrow V$ into a smooth variety *V*, then $\Gamma _{\mathcal {D}}(X,M) := {\text {Hom}}_{i^{\bullet }\mathcal {O}_V}(\mathcal {O}_X, i^{\bullet }i_\natural M)$, for *M* a D-module on *X* and $i_\natural M$ the associated right D-module on *V* supported on *i*(*X*). This is the subspace of the global sections of $i_\natural M$ as a sheaf on *V*, $\Gamma (X,M)$, which are scheme-theoretically supported on *i*(*X*), i.e., locally annihilated by the ideal sheaf of *X*. This produces an $\mathcal {O}(X)$-module, and by Kashiwara’s equivalence it does not depend on the choice of embedding.

For a general variety, we can define the global sections functor on D-modules by gluing the functor on affine varieties (which by definition embed into smooth varieties). This is well defined since, for $U_1 \subseteq U_2$ affine, $\Gamma _{\mathcal {D}}(U_1, M|_{U_1}) = \Gamma _{\mathcal {D}}(U_2, M|_{U_2}) \otimes _{\mathcal {O}(U_2)} \mathcal {O}(U_1)$. We therefore obtain an $\mathcal {O}(X)$-module, $\Gamma _{\mathcal {D}}(X,M)$, for an arbitrary variety *X*. In the case *X* embeds into a smooth variety, we recover the same answer as before (by restricting the embedding to affine subvarieties). We caution, however, that *even when* *X* *is affine, the global sections functor is not in general exact*.

Next, given any (not-necessarily smooth) variety *X*, there is a canonical quasi-coherent D-module, denoted by $\mathcal {D}_X$, such that ${\text {Hom}}(\mathcal {D}_X, N) = \Gamma _{\mathcal {D}}(X, N)$ for all D-modules *N* on *X*, i.e., $\mathcal {D}_X$ represents the functor of global sections. It may be defined as follows. Given any open (affine) subset $U \subseteq X$ and closed embedding $i: U \rightarrow V$ into a smooth variety, let $\mathcal {I}_U$ be the ideal sheaf of *i*(*U*). Then, we have the D-module $\mathcal {I}_U \cdot \mathcal {D}_V {\setminus } \mathcal {D}_V$ supported on *i*(*U*), and we may set $\mathcal {D}_U := i^\natural (\mathcal {I}_U \cdot \mathcal {D}_V {\setminus } \mathcal {D}_V)$. One may check explicitly that the definition does not depend on the choice of closed embedding and that ${\text {Hom}}(\mathcal {D}_U, N) = \Gamma _{\mathcal {D}}(U, N)$ for all D-modules *N* on *U*. Moreover, the definition is local: $\mathcal {D}_{U_1} = \mathcal {D}_{U_2}|_{U_1}$ for $U_1 \subseteq U_2$. We see therefore that these glue to a D-module, $\mathcal {D}_X$, on *X*. (Note that for quasi-projective varieties one need not worry about gluing, taking $U=X$.) Even when *X* is affine, this D-module is not, in general, projective, which explains why the global sections functor is not, in general, exact.

In the case that *X* is smooth, we can use the identity embedding $X \rightarrow X$ and consider D-modules to be modules over the ring of differential operators. In this case $\mathcal {D}_X$ identifies with the sheaf of differential operators on *X* as a right module over itself. In general, though, $\mathcal {D}_X$ is only given by an assignment to each open affine subset $U \subseteq X$ together with an embedding $U \rightarrow V$ into an affine space of a sheaf $\mathcal {D}_U$ on *V* (which by Kashiwara’s equivalence does not depend on the embedding); so we cannot compare $\mathcal {D}_X$ with the sheaf of rings of differential operators on *X*, as the two are different types of objects.

If *X* is affine, it is actually true, although nontrivial, that the global sections $\Gamma _\mathcal {D}(\mathcal {D}_X) = {\text {Hom}}(\mathcal {D}_X,\mathcal {D}_X)$ of $\mathcal {D}_X$ identify with Grothendieck’s ring of differential operators on *X*, but it is still not true that quasi-coherent D-modules are the same as right modules over this ring. In fact, for general singular affine varieties *X*, the category of D-modules on *X* need not be equivalent to the category of modules over any ring.

### Holonomic D-modules

#### Definition A.11

A coherent right D-module on a smooth affine variety *V* is a finitely-generated quasi-coherent right $\mathcal {D}(V)$-module. A coherent D-module *M* on an arbitrary affine variety *X* is one such that, for any (equivalently every) closed embedding $i: X \rightarrow V$ into a smooth affine variety, the corresponding D-module on *V* supported on *i*(*X*) is coherent. A coherent D-module *M* on an arbitrary variety *X* is one such that the restriction of *M* to every open affine subset $U \subseteq X$ is coherent.

Now recall that if *V* is a smooth affine variety, then the ring $\mathcal {D}(V)$ of differential operators is equipped with a filtration $\mathcal {D}(V) = \bigcup _{m \ge 0} \mathcal {D}_{\le m}(V)$ by order of operator such that ${\textsf {gr}}(\mathcal {D}(V)) := \bigoplus _{m \ge 0} \mathcal {D}_{\le m}(V) / \mathcal {D}_{\le (m-1)}(V)$ is identified with the algebra $\mathcal {O}(T^* V)$ of functions on the total space of the cotangent bundle $T^* V$ of *V*.

#### Definition A.12

Given a quasi-coherent right D-module *M* on a smooth affine variety *V*, a good (nonnegative) filtration is an filtration $M_{\le 0} \subseteq M_{\le 1} \subseteq \cdots$ of subsets of *M* which is exhaustive ($M = \bigcup _{m \ge 0} M_{\le m}$), with $M_{\le m} \mathcal {D}_{\le n} \subseteq M_{\le n}$ for $m,n \ge 0$, and such that ${\textsf {gr}}M = \bigoplus _{m \ge 0} M_{\le m} / M_{\le m-1}$ is a finitely-generated ${\textsf {gr}}\mathcal {D}(V) = \mathcal {O}(T^*V)$-module (with $M_{\le -1} := 0$).

As explained in, e.g., [36, Theorem 2.13], every coherent right D-module *M* admits a good filtration: let $M_{\le 0}$ be any finite-dimensional subspace which generates *M*, and set $M_{\le m} := \mathcal {D}(V)_{\le m} M_{\le 0}$ for all $m > 0$. Conversely, all quasi-coherent D-modules admitting good filtrations are coherent.

#### Definition A.13

Given a coherent right D-module *M* on a smooth affine variety *V* equipped with a good filtration, the characteristic variety, ${\textsf {Ch}}(M)$, is defined to be the set-theoretic support of ${\textsf {gr}}M$ over $T^* V$.

One can check that the characteristic variety does not depend on the choice of good filtration. (This follows from [36, Theorem 2.13].) This allows one to extend the notion of characteristic variety to the not-necessarily affine case, since the characteristic variety is defined locally. We conclude that every coherent right D-module on a smooth variety has a well-defined characteristic variety.

#### Definition A.14

A (nonzero) holonomic right D-module on a smooth irreducible variety *V* is a coherent right D-module whose characteristic variety has dimension equal to the dimension of *V*.

In fact, the characteristic variety is well known to be a coisotropic subvariety of $T^* V$ (by a theorem of Sato, Kawai, and Kashiwara [55]; see also [26]), so the (nonzero) D-module is holonomic if and only if this is Lagrangian (and hence has the minimal possible dimension). By convention, the zero module is also holonomic.

### Direct and inverse image

Given a map $f: X \rightarrow Y$ of smooth varieties, we have natural functors $f^!: D^b(\text {mod}-\mathcal {D}_Y) \rightarrow D^b(\text {mod}-\mathcal {D}_X)$ and $f_*: D^b(\text {mod}-\mathcal {D}_X) \rightarrow D^b(\text {mod}-\mathcal {D}_Y)$. These are not, in general, the derived functors of any functors on abelian categories. However, when *f* is affine, $f_*$ is the derived functor of a right exact functor, and when *f* is an open embedding, $f_*$ is the derived functor of a left exact functor (and in this case $f^!$ is the exact restriction functor). In particular, if *f* is a closed embedding, then $f_*$ is the derived functor of the exact functor $f_\natural$ (now viewed as having target equal to all quasi-coherent D-modules on *Y*). Also, when *f* is a closed embedding, $f^!$ is the derived functor of a left exact functor (which is given by the same definition as $f^\natural$, now defined on all $\mathcal {D}$-modules on *Y* rather than merely those set-theoretically supported on *f*(*X*)). In particular, when *f* is a closed embedding, $f_*$ coincides with $f_\natural$ (more precisely, $f_\natural M$ is the cohomology of $f_* M$), and $f^!$ coincides with $f^\natural$ on $\mathcal {D}$-modules supported on *f*(*X*) (more precisely, $f^\natural M$ is the cohomology of $f^! M$ if *M* is supported on *f*(*X*)). The definitions are:

A.3 $$\begin{aligned} f_* M&:= Rf_\bullet \left( M \otimes ^L_{\mathcal {D}_X} \mathcal {D}_{X \rightarrow Y}\right) , \quad \mathcal {D}_{X \rightarrow Y} := \mathcal {O}_X \otimes _{f^\bullet \mathcal {O}_Y} f^{\bullet } \mathcal {D}_Y; \end{aligned}$$

A.4 $$\begin{aligned} f^!(M)&:=f^\bullet (M) \otimes ^L_{f^\bullet \mathcal {D}_Y} \mathcal {D}_{Y \leftarrow X}[\dim X - \dim Y], \nonumber \\ \mathcal {D}_{Y \leftarrow X}&:= \Omega _X \otimes _{\mathcal {O}_X} \mathcal {D}_{X \rightarrow Y} \otimes _{f^\bullet \mathcal {O}_Y} f^\bullet \Omega _Y^{-1}. \end{aligned}$$

See [36, Sect. 1.3, 1.5].

#### Theorem A.15

(e.g., [36, Theorem 3.2.3]) Let $f: X \rightarrow Y$ be a map of smooth varieties and *M* and *N* bounded complexes of quasi-coherent right D-modules on *X* and *Y* whose cohomology D-modules are holonomic. Then $f^! N$ and $f_* M$ are bounded complexes whose cohomology D-modules are holonomic.

#### Corollary A.16

Let *X* be an arbitrary variety and *M* a quasi-coherent D-module on *X*. Then, given two closed embeddings $i_1: X \rightarrow V_1$ and $i_2: X \rightarrow V_2$, $(i_1)_{\natural } M$ is holonomic if and only if $(i_2)_{\natural } M$ is.

Therefore, we can make the following definition:

#### Definition A.17

A quasi-coherent D-module *M* on an affine variety *X* is called holonomic if, for any closed embedding $i: X \rightarrow V$ into a smooth affine variety, $i_\natural M$ is holonomic. A D-module *M* on an arbitrary variety *X* is called holonomic if, for every open affine subset $U \subseteq V$, the restriction $M|_U$ of *M* to *U* is holonomic.

With this definition in place, Theorem A.15 immediately generalizes to arbitrary varieties. Namely, if $X \rightarrow Y$ is an arbitrary map of varieties, then one obtains canonical functors $f^!: D^b(\mathcal {D}-\text {mod}_Y)\rightarrow D^b(\mathcal {D}-\text {mod}_X)$ and $f_*: D^b(\mathcal {D}-\text {mod}_X)\rightarrow D^b(\mathcal {D}-\text {mod}_Y)$ preserving holonomicity:

#### Corollary A.18

Let $f: X \rightarrow Y$ be an arbitrary map of varieties and *M* and *N* bounded complexes of quasi-coherent D-modules on *X* and *Y* whose cohomology D-modules are holonomic. Then, $f^! N$ and $f_* M$ are bounded complexes whose cohomology D-modules are holonomic.

Observe that when *X* is a point, a holonomic D-module is merely a finite-dimensional vector space (since finite generation reduces to finite dimensionality, and the support condition is trivial). We therefore deduce:

#### Corollary A.19

If *M* is a complex of quasi-coherent D-modules on a variety *X* with holonomic cohomology and $\pi : X \rightarrow \text {pt}$ is the projection, then $\pi _* M$ is a complex with finite-dimensional cohomology. In particular, if *M* is a holonomic D-module, then $H^0\pi _{*} M$ is a finite-dimensional vector space.

## Footnotes

1 For a proof that the conjecture above is the same as the statement from [23], see Proposition 3.13 of the arXiv version 1401.5042 of [23], and apply Grothendieck–Serre duality to identify $H^{\dim Y}(Y,\mathcal {O}_Y)$ with global sections of the logarithmic canonical bundle on *Y*. See also the second paragraph of the proof of [14, Lemma 2.5].
